# CyberRoBERTa: A Lightweight Transformer-Based Language Model for IoT Intrusion Detection

**DOI:** 10.3390/s26196326

**Published:** 2026-10-07

**Authors:** Dhai Alhafi, Fatmah Alanazi

**Affiliations:** Computer Science Department, College of Computer and Information Sciences, Imam Mohammad Ibn Saud Islamic University (IMSIU), Riyadh 11432, Saudi Arabia; 445018374@sm.imamu.edu.sa

**Keywords:** domain-specific language model, Internet of Things (IoT), intrusion detection systems, masked language modeling (MLM), small language models, tiny language models, transformer-based intrusion detection, Unigram tokenizer

## Abstract

With the accelerated proliferation of the Internet of Things (IoT) and its widespread use in sensitive areas such as healthcare, homes, and factories, security has become an increasingly critical concern. IoT remains one of the most challenging domains to secure, lacking the manageable scale, well-maintained software, and homogeneous environments of easily secured systems. Large language models (LLMs) have recently been applied to improve IoT security; however, most applications treat LLMs as “off-the-shelf” tools, creating a domain mismatch because model representations, tokenizers, and data collators are designed for natural language rather than network traffic. In this work, we present CyberRoBERTa, built specifically to test whether departing from off-the-shelf LLM components, through a domain-specific Unigram tokenizer and an IoT-specialized data collator, yields a genuine advantage for IoT intrusion detection. We instantiate this framework as a compact, 10.2-million-parameter model designed for fully self-contained, on-device operation. Using CICIoT2023, TON_IoT, and Edge-IIoTset, the model achieves a binary classification F1-score of 96% and a multiclass F1-score of 89.54%. We further validate these results in three ways: a comparison against a classical machine learning baseline shows comparable accuracy; isolating the tokenizer’s contribution from pretraining scale shows its benefit holds under matched pretraining conditions; and evaluating class-imbalance interventions shows improved detection on the most data-scarce class.

## 1. Introduction

The emergence of the Internet of Things (IoT) has profoundly altered the technological landscape. Coined in the late 1990s, the term IoT describes a system that connects the digital world with the physical world through vast networks of interconnected devices and sensors capable of communicating, collecting data, and making decisions. IoT has improved the overall quality of life across a wide range of sectors. For instance, IoT has become an essential paradigm for smart cities, ensuring efficient resource utilization through large-scale sensing, real-time data gathering, and intelligent decision-making in heterogeneous urban systems [1]. Additionally, IoT has been utilized in healthcare to improve patient care, offering remote monitoring, personalized treatment plans, and efficient healthcare delivery [2]. Furthermore, industrial IoT, known as IIoT, is considered a cornerstone of Industry 4.0, enabling predictive maintenance, real-time supply chain visibility, and overall enhancement of manufacturing automation [3]. The benefits of IoT continue to expand into homes; for instance, smart cameras and voice-activated assistants emphasize user convenience and efficiency [4]. These advantages have driven IoT’s rapid proliferation and globalization. According to IoT Analytics, the number of connected devices reached 18.5 billion in 2024, with an estimated growth rate of 14% expected to bring that number to 21.1 billion by the end of 2025, though the actual 2025 total may fall short of this forecast by roughly 300 million connections [5]. Nevertheless, the rapid growth of IoT underscores its significant potential across many fields, while simultaneously highlighting new challenges, especially in the realm of cybersecurity.

With the accelerated distribution and implementation of IoT worldwide, security has become a major concern. Despite its positive impact, IoT remains insecure for several reasons; for instance, IoT is built to be small, efficient, and fast, leaving security as an afterthought [6]. Furthermore, IoT devices generate billions of heterogeneous data points daily, ranging from home videos to industrial operational data to sensitive health records. This creates a vast and complex attack surface [7]; hence, as adversaries invariably evolve, a robust yet lightweight solution is needed to address these concerns.

Signature-based detection, the traditional foundation of intrusion detection systems, identifies attacks by matching traffic against predefined rules. It offers high accuracy, low false-positive rates, and transparent decision-making for known threats, but it cannot detect zero-day attacks, is easily evaded through minor changes to an attack’s signature, and requires constant signature-database maintenance, leaving it blind to multi-phase attacks. Machine learning (ML) and deep learning (DL) emerged to address these gaps, offering the ability to generalize beyond known signatures. However, ML and DL models introduce their own limitations: many DL architectures, particularly recurrent designs, process data sequentially rather than in parallel, limiting their ability to detect long-range attacks such as Advanced Persistent Threats (APTs), and despite decades of development, NN-based detection methods continue to face significant challenges achieving reliable performance in practical deployment, as NN-based detection systems that perform well in controlled laboratory evaluations frequently miss real attacks once deployed against real-world traffic conditions [8,9]. Because of the limitations of ML and DL, large language models (LLMs) are emerging as a solution across multiple sectors, such as healthcare, customer service, and content creation and marketing [10]. LLMs have also demonstrated promising results in other domains; for instance, in cybersecurity, these models are implemented in penetration testing, intrusion detection systems (IDS), and system-log analysis [11]. Nevertheless, existing IoT intrusion detection literature has two main gaps as of today. First, much of this work relies on LLMs whose scale is difficult to justify for deployment on or near IoT devices. [12] combined BERT and GPT-2, together exceeding 180million parameters, in a proactive attack-prediction pipeline. Furthermore, [13] applied a 110-million-parameter BERT-based model to intrusion detection in the Internet of Vehicles. Models of this scale, roughly ten to twenty times larger than the model proposed in this paper, remain difficult to reconcile with the memory and power constraints of typical IoT hardware.

Second, recent research treats LLMs as “off-the-shelf” solutions, applying them largely unmodified regardless of dataset needs, model size, or tokenizer design. Even work that reduces model size does not necessarily adapt the tokenizer or pretraining pipeline:  [14] proposed a lightweight BERT-based model for IIoT anomaly detection, but they relied on Privacy-Preserving Fixed-Length Encoding (PPFLE) to represent network traffic rather than a tokenizer designed for it, leaving BERT’s original natural language pretraining unchanged. Ref. [15] fine-tuned general-purpose T5-small and T5-base checkpoints directly on intrusion detection data without adapting the tokenizer or architecture to the structure of network traffic, and the authors reported overfitting as a result. Ref. [16] reduced the number of BERT’s transformer layers for IoT deployment but retained BERT’s original WordPiece tokenizer, trained on natural language corpora, without adaptation to network-specific vocabulary. This pattern suggests that current IoT intrusion detection research treats tokenization and pretraining largely as fixed inheritances from natural language processing, rather than as design choices to be revisited for network-traffic data. Taken together, this strategy assumes that tokenization and pretraining approaches optimized for natural language (NL) are equally suitable for the highly repetitive, number-containing structure of network flows. To address these critical gaps, this paper makes the following contributions:

1. A domain-specific masking strategy for network traffic. We introduce CyberValueCollator, a masked language modeling collator that separates constant schema tokens from variable behavioral values and corrupts only the latter under an aggressive 10-10-80 scheme. Unlike conventional MLM, which masks tokens indiscriminately, this design forces the model to learn IoT network behavior directly from traffic statistics rather than memorizing fixed feature vocabulary. We further evaluate the mechanism governing how corrupted values are replaced, finding that feature-constrained alternatives to the default random-substitution scheme substantially improve detection of the most data-scarce attack class evaluated.
2. A Unigram-based cyber tokenizer purpose-built for network flows. We design a domain-specific tokenizer that replaces conventional subword tokenization with a probabilistic Unigram model and injects all 57 network-flow feature names as locked, atomic tokens. This prevents the fragmentation that generic, natural language tokenizers impose on structured, numerically dense traffic data, yielding a vocabulary that reflects the actual semantics of network flows. We isolate this tokenizer’s contribution from sequence truncation and pretraining scale, finding a genuine accuracy advantage under matched pretraining conditions, the realistic scenario for a domain-specific model trained from scratch.
3. A compact, efficient language model for IoT intrusion detection. By pairing these two innovations with a deliberately compact RoBERTa configuration, we obtain a 10.2-million-parameter model that achieves competitive detection performance while remaining practical for deployment in constrained IoT environments.
4. A masking-rate ablation study for domain-specific pretraining. We evaluate CyberValueCollator across four masking rates (15%, 20%, 25%, and 30%), alongside a standard 15% baseline, to determine an appropriate masking intensity for network traffic rather than adopting a fixed rate by default. The results show that masking rate materially affects downstream detection performance, particularly on the classes hardest to detect, supporting the treatment of masking strategy as a domain-specific design choice.
5. Serialized IoT traffic corpora built for pretraining an intrusion-detection language model. We construct two corpora specifically for this purpose: an unlabeled pretraining corpus drawn from the normal-traffic partitions of CICIoT2023 and TON_IoT, and a labeled fine-tuning corpus built from Edge-IIoTset with both binary and multiclass attack labels. This unlabeled/labeled split allows the encoder to learn general IoT network behavior during pretraining before being explicitly supervised on attack detection during fine-tuning. Both corpora, together with the pretrained model weights, are released to support reproducibility.
6. Comprehensive empirical validation of design choices and deployment feasibility. We evaluate CyberRoBERTa against a classical machine learning baseline on identical features, isolate the tokenizer’s contribution from sequence truncation and pretraining scale, verify feature-space overlap for the hardest-to-detect classes independent of any trained classifier, evaluate four interventions targeting class imbalance, and provide an empirical and theoretical analysis of inference latency, memory, and computational cost, including a projection onto constrained edge hardware.

The rest of the paper is organized as follows: Related Work, Methodology, Results, Discussion, and Conclusion.

## 2. Related Work

### 2.1. Domain Adaptation Through Customized Tokenization

As language models move into domain-specific fields such as medical care, legal documents, and cybersecurity, questions of applicability and suitability naturally arise. In the legal domain, for example, archaic and highly specific terminology often defeats general-purpose tokenizers, which tend to split such words into meaningless subwords. Ref. [17] addressed this by introducing the KL3M family of tokenizers for legal, financial, and governmental text; the tokenizers were evaluated in terms of efficiency and domain-term representation, and KL3M achieved better results than a general-purpose tokenizer. Similarly, [18] introduced “clinical tokens” by augmenting the vocabulary of the original LLaMA2 tokenizer, keeping medical terms intact and enabling the model to learn more effectively from clinical data. However, while these studies explore various tokenization methods, only the MIND-IoT framework introduced by [19] targets IoT network traffic specifically, addressing IoT traffic classification tasks such as device-type and category identification rather than intrusion detection. MIND-IoT’s tokenizer excludes a set of predefined feature-name tokens from subword fragmentation during training, using Byte Pair Encoding as its segmentation algorithm. We adopt and extend this locked-token approach for intrusion detection settings.

### 2.2. Advanced Masking Strategies and Custom Data Collation

Large language model (LLM) training has been greatly influenced by masked language modeling (MLM), the approach underlying bidirectional models such as BERT and RoBERTa. In MLM, a subset of tokens is hidden from the model, which must then predict the missing values using context from the rest of the sequence. MLM was introduced with BERT using a conventional masking rate of 15%, which has since become standard practice when training new models. Nevertheless, more recent work has challenged this fixed rate: [20] found that while 15% masking is effective, it is not optimal, since larger models benefit from more aggressive masking rates on benchmarks such as GLUE and SQuAD. Ref. [21] followed a similarly aggressive approach in developing a more efficient encoder. However, both studies focus on English-language text and are not domain-specific. Within the IoT domain specifically, [19] apply the standard MLM objective with a flat 15% masking rate uniformly across all tokens, without distinguishing between fixed feature-name tokens and variable behavioral values. This leaves open the question of whether a masking strategy tailored to the structural distinction between schema and behavioral tokens could further improve representation learning for network traffic, a gap we address directly with CyberValueCollator.

### 2.3. Machine Learning, Deep Learning, and Transformer-Based Anomaly Detection Systems

Machine learning has been widely applied to cybersecurity. Ref. [22] used multiple machine learning models, including Decision Tree (DT), Random Forest (RF), Logistic Regression (LR), Extreme Gradient Boosting (XGB), and K-Nearest Neighbors (KNN) to build a real-time intrusion detection system capable of detecting different attack types. Each model was trained and evaluated separately on the same dataset using metrics such as accuracy, recall, F1-score, precision, and training time; results varied across models, with XGB achieving the highest accuracy at 98.89%. Despite these results, each method carried its own limitation; logistic regression, for example, struggled to classify benign traffic accurately. Ref. [23] proposed a hybrid intrusion detection system for smart buildings that combines XGB with principal component analysis (PCA), designed and tested on an Orange Pi to suit computationally constrained devices; however, its dependence on smart-home datasets may limit its performance in other IoT environments. Deep learning offers another approach to intrusion detection. Ref. [24] introduced VGGIncepNet, which converts network data into 2-D images before applying a CNN for anomaly detection; while VGGIncepNet outperformed other deep learning baselines, it demands high computational resources and generalizes poorly. Ref. [25] developed DS-SIoDL, an intrusion detection system combining long short-term memory (LSTM) networks with feature engineering techniques such as autoencoders (AE), information gain (IG), and a genetic algorithm (GA); the model achieved strong results, though because LSTM processes data sequentially rather than in parallel, it faces a design limitation that affects both efficiency and its ability to detect Advanced Persistent Threats (APTs). Machine learning and deep learning algorithms generally offer fast inference times and high interpretability, and perform well on structured, tabular network data with well-defined features. Nevertheless, these models lack the self-attention mechanism central to transformer-based approaches, which allows the model to capture global dependencies across an entire sequence simultaneously, regardless of distance; transformer-based models are also highly parallelizable and excel at learning complex contextual relationships in network protocol sequences.

Large language models (LLMs) have also proven their feasibility in security. Ref. [14] introduced SecurityBERT, an intrusion detection system that pairs BERT with Privacy-Preserving Fixed-Length Encoding (PPFLE) to detect anomalies in IIoT while preserving privacy; it outperformed traditional machine learning and deep learning baselines, though it was evaluated on only one dataset, raising concerns about generalization. Ref. [12] proposed a proactive intrusion prediction system that combines BERT, GPT, and LSTM to predict attacks before they occur: GPT is fine-tuned to predict the next network packet, BERT evaluates GPT’s predictions, and LSTM classifies the resulting traffic as normal or malicious. This approach goes beyond detection by predicting attacks before they occur; however, combining computationally intensive models such as BERT and GPT imposes a heavy toll on IoT, particularly when deployed on or near IoT devices. Moreover, despite using the CICIoT2023 dataset, which covers thirty-three attack types, the authors evaluated only five, limiting the demonstrated generalizability of their approach. Ref. [13] extended LLM-based intrusion detection to the Internet of Vehicles (IoV), introducing IoV-BERT-IDS to detect attacks originating from both within and outside IoT vehicles; the system outperformed other deep learning models, though implementing BERT remains computationally intensive. Ref. [15] applied two versions of the T5 language model, T5-small and T5-base, models designed primarily for text and language tasks, to intrusion detection; their resulting system surpassed traditional machine learning models, reaching 99% accuracy, though limited training data combined with the models’ complexity led to overfitting, and T5-base remains computationally intensive. Ref. [16] introduced a BERT-based intrusion detection system that operates at both the network and application layers, optimized for IoT by reducing BERT’s architecture: the application-layer IDS uses only 7 of BERT’s 12 layers. In comparison, the network-layer IDS retains all 12 layers but with fewer parameters per layer. Despite these strong results, the paper does not explain why it chose seven layers, nor does it discuss any resulting drawbacks. A natural point of comparison is [26]’s SEED framework, which addresses the same deployment constraint through a different strategy. Rather than shrinking a single model to run entirely on-device, SEED partitions the workload across two tiers: a 40.6 MB BERT model (10,154,767 parameters) hosted at the network edge generates semantic embeddings of network traffic, while a compact 137 KB classifier on the IoT device queries those embeddings to make the final decision. SEED reports 9 ms edge-side embedding generation and 70 ms IoT-side classification latency on a 2.5 GHz CPU, with near-perfect accuracy across a 15-class Edge-IIoTset evaluation. Its edge-side model is close in parameter count to CyberRoBERTa’s complete, self-contained model (10,230,414 parameters, 39.03 MB). SEED’s feature encoding differs from ours in both purpose and design: values are anonymized through a privacy-preserving hash prior to tokenization, whereas CyberRoBERTa instead locks feature names as atomic tokens specifically to preserve their semantic identity, reflecting two different priorities: privacy-preserving anonymization versus domain-semantic preservation. Beyond footprint and accuracy, the two architectures differ in a way with direct security implications: SEED’s reliance on continuous embedding transmission between the IoT device and the edge server introduces a network-dependent attack surface that CyberRoBERTa’s fully self-contained, single-device design avoids by construction. This is not a hypothetical concern; SEED’s own authors note that transmitted embeddings “may be susceptible to interception or reverse engineering, potentially revealing sensitive device metadata,” identifying this as an open limitation. For an intrusion detection system specifically, this carries a notable implication: a detector whose operation depends on an external communication channel is potentially exposed to the same class of interception-based attacks it is designed to detect. Furthermore, [27] demonstrate a structurally similar argument in an adjacent domain: adversarial-detection methods designed for other fields (e.g., image-based reconstruction detectors) underperform when applied directly to deep learning-based soft sensors, motivating a bidirectional, domain-specific detection architecture (BiLSTM-KAN) tailored to the temporal continuity of industrial process data. Notably, their comparison baselines include an XGBoost-based detector, which—like the XGBoost baseline in this work Section 4.5—degrades sharply on the most stealthy or camouflaged case in its benchmark, while the domain-adapted method does not. This parallel reinforces a broader pattern across application domains: naively transplanted methods tend to fail precisely on the hardest cases, while domain-specific redesign narrows that gap. CyberRoBERTa extends this principle to a domain not addressed by this paper: discrete, tokenized network-flow data processed by transformer-based language models, where the relevant redesign occurs at the level of tokenization and the pretraining masking objective rather than output-consistency checking. Ref. [28] proposed an intrusion detection approach based on attention-based Transformer architectures, trained entirely from scratch on correlation-selected numeric features rather than adapted from a pretrained natural language model, achieving 99% accuracy in both binary and multiclass classification with a notably compact 13,718-parameter architecture. Nevertheless, like other prior work discussed here, this study applies its architecture without adaptation to the structural properties of network-flow data, specifically, the schema-versus-behavioral-value distinction central to CyberValueCollator’s design. The specific mechanism by which corrupted values are replaced has also been studied outside the natural language setting, particularly for tabular and structured data. Ref. [29] found that, for tabular representation learning, naively sampling replacement values at random from the full vocabulary is comparatively ineffective, since such substitutions are often trivially distinguishable from genuine values by local statistical cues; their TABBIE model instead adopts intra-table cell swapping, drawing replacement values from other entries within the same column, and reports that this column-aware corruption strategy yields stronger learned representations than vocabulary-wide random sampling. A related distinction appears in the broader denoising-autoencoder literature for tabular data, where corruption strategies are commonly categorized as “swap noise” (replacing a value with another real value drawn from the same feature’s distribution) and “zero noise” (replacing a value with a fixed placeholder), both of which are less likely than unconstrained random substitution to introduce combinations inconsistent with the underlying feature’s marginal distribution. CyberValueCollator’s default configuration instead draws replacement values from across the full training vocabulary regardless of feature identity; Section 4.1.2 evaluates this choice directly against feature-constrained (swap-noise-style) and zero-replacement alternatives to determine whether the column-aware corruption principle established for general tabular data also holds for serialized network-flow records.

## 3. Methodology

### 3.1. Dataset Preparation and Selection

The proposed model is trained and evaluated on three datasets: CICIoT2023, TON_IoT, and Edge-IIoTset.

#### Data Collection and Selection

Three datasets are selected according to the following conditions:

1. The dataset must be recent, as the IoT attack landscape evolves quickly and a current dataset is required to reflect contemporary attack types.
2. The dataset must be published in a peer-reviewed venue, which reduces the reproducibility and credibility risks associated with unvetted collections.
3. The dataset must be comprehensive, covering normal traffic, attack traffic, and multiple attack categories simultaneously.

Further conditions were applied to ensure the quality of encoder training. The pretraining corpus must be unlabeled, since it is used only to teach the model IoT traffic representations; this ensures no leakage or memorization during pretraining. We additionally restrict pretraining to normal traffic only, a choice worth distinguishing from anomaly-detection approaches, where training exclusively on normal traffic is a structural requirement: a model trained to reconstruct normal behavior would begin treating attack patterns as expected, rather than anomalous, if exposed to them during training. CyberRoBERTa’s objective works differently. Masked language modeling never uses traffic labels, and the model is never asked to distinguish normal from malicious behavior during pretraining, only to reconstruct masked values from context. Under this objective, restricting the corpus to normal traffic is a design choice rather than a requirement, since the model would not learn to recognize attacks from unlabeled attack traffic regardless of whether it were included. Instead, we aimed to build a general understanding of IoT network behavior, unbiased by our source datasets’ specific staged attack scenarios; we learn attack detection entirely during fine-tuning, where the model is directly supervised on labeled examples from Edge-IIoTset. The fine-tuning corpus is constructed from 19 raw traffic files: 11 attack files and 8 normal-traffic files, labeled for both binary and multiclass classification. This choice is not without cost: since the encoder never observes attack traffic during pretraining, it enters fine-tuning with no prior representation of attack-specific feature-space regions, placing the full burden of learning attack behavior on the comparatively small labeled fine-tuning corpus. We revisit this limitation, and its connection to specific classes’ detection ceilings, in Section 6.

CICIoT2023, published in 2023, is a comprehensive IoT dataset covering 33 attack types across 105 devices, released by the Canadian Institute for Cybersecurity (CIC). The dataset is designed specifically for intrusion and anomaly detection research and reflects real-world IoT traffic through a realistic testbed simulating communication among multiple IoT devices, including smart TVs, webcams, smart thermostats, and wearable devices, capturing both benign and malicious traffic [30]. TON_IoT is a comprehensive, realistic dataset comprising heterogeneous data sources collected from IoT and IIoT sensors, gathered from a network designed at the Cyber Range and IoT Labs of the School of Engineering and Information Technology (SEIT). TON_IoT also includes operating-system datasets for Windows 7 and 10, as well as Ubuntu 14 and 18, along with TLS and network traffic data [31]. Although both TON_IoT and CICIoT2023 cover multiple attack types, we use only their normal traffic during pretraining.

Edge-IIoTset is used for fine-tuning. It is a realistic, comprehensive cybersecurity dataset covering both IoT and IIoT, built using wireless routers, a Raspberry Pi 4 Model B, ESP32 and Arduino Uno boards, sensors and actuators, desktop computers, smartphones, and smart TVs. Edge-IIoTset covers 15 attack types, including DoS/DDoS, information gathering, man-in-the-middle (MITM), injection, and malware attacks [32]. Table 1 summarizes the selected datasets.

### 3.2. Feature Extraction and Text Serialization

Our feature extraction and text serialization approach builds on the custom IoT-Tokenize pipeline proposed by [19], adding a new scaling step and constructing labeled and unlabeled corpora for cybersecurity-focused language model pretraining and fine-tuning. Raw network pcap files were collected from their respective sources and processed using the NFStream library [33], a Python-based analyzer (Python 3.13.15, NFStream 6.6.0) that performs bidirectional flow aggregation and extracts features suitable for machine learning. NFStream was configured with statistical_analysis=True across all datasets, enabling computation of inter-arrival time distributions and packet size moments beyond basic packet and byte counts. The pretraining extraction script processed 18 pcap files of normal traffic only. Moreover, NFStream is utilized recursively without a per-file flow limit, yielding a combined 197 MB pretraining corpus drawn from CICIoT2023 and TON_IoT, as summarized in Table 1. The fine-tuning extraction script processed pcap files the same way, yielding a 66.8 MB fine-tuning corpus from Edge-IIoTset (Table 1), with an added labeling step: a binary label (0 for normal, 1 for attack) and an attack-class label identifying the specific attack category. In total, 11 attack types from Edge-IIoTset were used for fine-tuning and evaluation.

During extraction, several features were dropped to prevent feature leakage, that is, cases in which a feature carries information about the target variable that would not be available at inference time, causing the model to learn spurious, dataset-specific correlations rather than genuine network behavior. Therefore, we removed the following: first, pure identifiers, which reflect dataset structure rather than attack behavior; second, machine-identity features, such as IP and MAC addresses, which would let the model memorize fixed attacker addresses in lab settings rather than generalize to new hosts; third, timestamp features, which encode when attacks were injected and could let the model exploit the fixed attack window rather than learn the attack pattern itself; fourth, tool-specific signals, such as fixed JA3 hashes, empty SNI fields, and generic user-agent strings, which reflect the attack tool rather than the underlying attack semantics; and fifth, Deep Packet Inspection (DPI)-derived features, which may directly or indirectly encode the ground-truth label. The dropped features are listed in Table 2.

**Figure 1:**
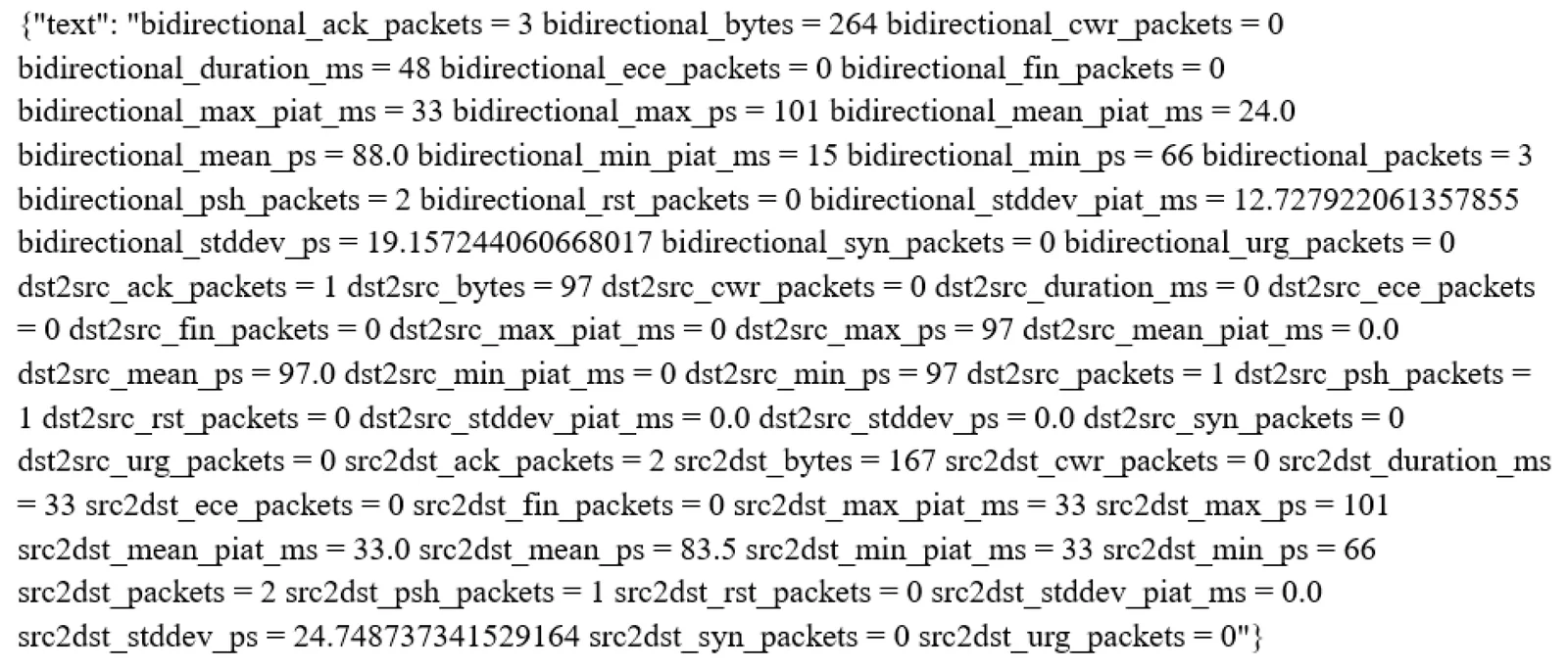
Sample from the pretraining dataset.

The remaining retained features were serialized into lines of text as header-value strings, with each line representing a single IoT network flow. This design makes the datasets suitable for tokenizer training and for model pretraining and fine-tuning using standard language-modeling tooling. The 57 retained features, which reflect IoT network behavior without leaking label information, are listed in Table 3. Moreover, a sample from the pretraining dataset is shown in Figure 1.

### 3.3. Data Cleaning and Scaling

Each dataset underwent a consistent cleaning process after extraction. There was one exception: the fine-tuning and testing datasets retained duplicate records for denial-of-service (DoS) and distributed denial-of-service (DDoS) attacks, since these attacks characteristically produce near-identical flows, and removing such duplicates would strip out the attack behavior itself. The pretraining dataset, by contrast, had duplicates removed to preserve training quality.

Because the datasets are converted into a text-like format, cleaning was carried out in three sequential validation steps. First, each record was checked to confirm it parses as valid JSON; malformed records were discarded. Second, each record was checked for its required fields: the text field alone for the pretraining corpus, and the text, binary label, and attack label fields for the fine-tuning corpus. Third, the text field was checked to ensure it was non-empty and non-null. Any record failing one of these checks was discarded.

The final processing step was feature scaling, applied to give the encoder a normalized numerical representation for training. Scaling was implemented in three stages: scaler selection by feature type, scaler fitting on the pretraining corpus, and application of the fitted scaler across the remaining datasets. Rather than apply a single uniform scaling method to all features, we adopted a feature-adaptive strategy based on each feature’s name and statistical properties. Features describing byte counts, duration, inter-arrival time, and packet-size extremes (bytes, duration, piat, ps, max, min) were assigned a RobustScaler, appropriate for the heavy-tailed distributions common in network traffic. Features representing distributional summaries, mean and standard deviation, were assigned a StandardScaler, since such values are already averaged and exhibit less extreme outlier behavior. Features counting discrete packet-flag events (ACK, SYN, FIN) were assigned a MinMaxScaler, mapping each value into the [0, 1] range. The scaler was fitted exclusively on the CICIoT2023 and TON_IoT pretraining corpus, and the resulting fitting parameters were applied to the Edge-IIoTset dataset.

### 3.4. Framework Overview

**Figure 2:**
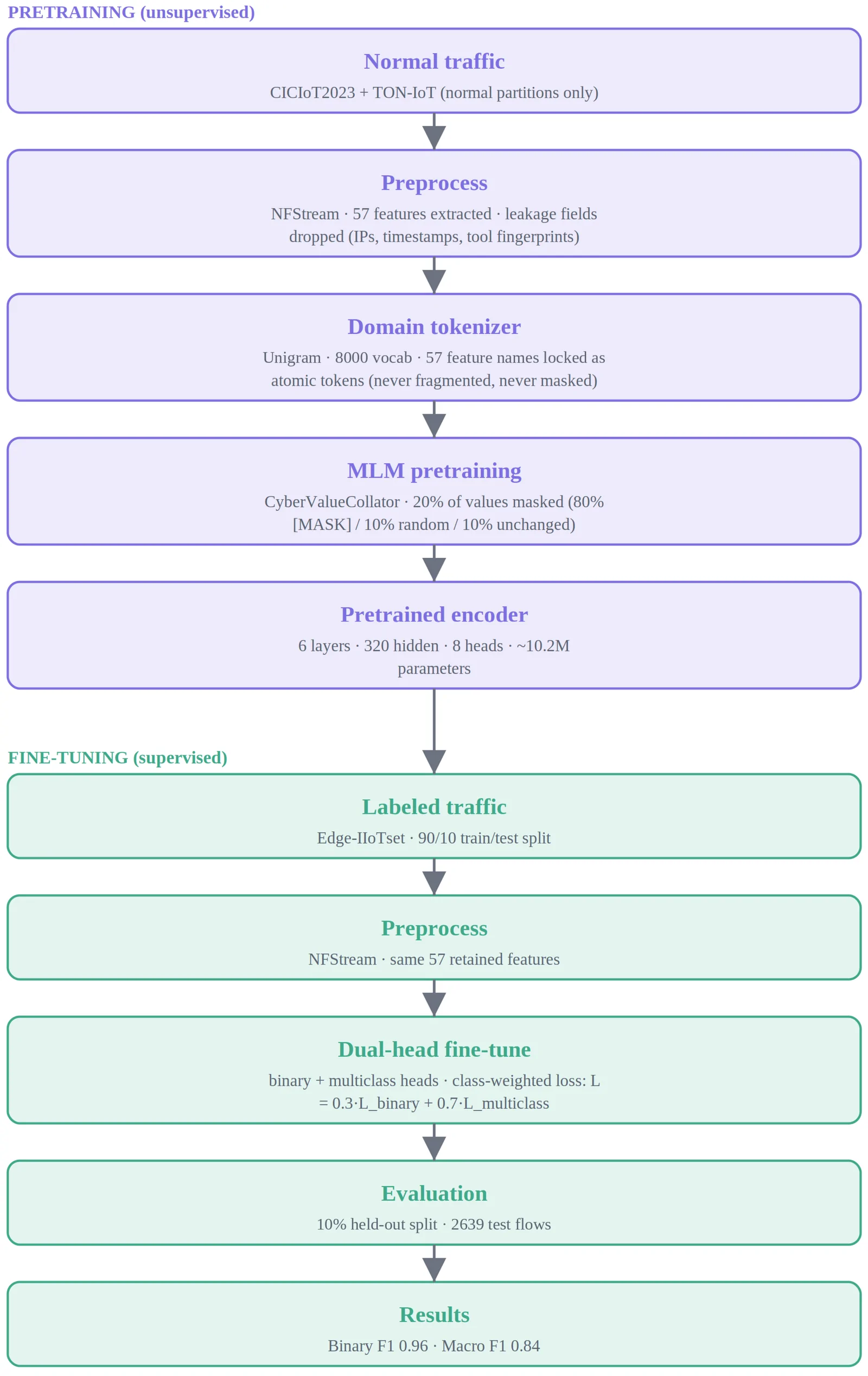
Overview of the CyberRoBERTa pretraining and fine-tuning pipeline.

The architectural decisions behind CyberRoBERTa are described in this section, including the domain-specific tokenizer, the pretraining encoder, the multitask fine-tuning heads, and the domain adaptation strategy. The complete framework is illustrated in Figure 2.

The pipeline begins with raw pcap processing, converting each of the three datasets, CICIoT2023, TON_IoT, and Edge-IIoTset, into cleaned, scaled, serialized flow records via NFStream. CICIoT2023 and TON_IoT are used exclusively in their normal-traffic partitions to construct the pretraining corpus. In contrast, Edge-IIoTset, used in both its normal and attack partitions, serves as the supervised fine-tuning corpus. The result is two textual datasets, each built from the same 57 retained features. The CyberRoBERTa encoder follows the RoBERTa architecture with a domain-specific configuration: 6 transformer layers, a hidden size of 320, 8 attention heads, and an intermediate feedforward size of 1280, totaling 10.2 million parameters. All weights are initialized from scratch and pretrained on the normal traffic from CICIoT2023 and TON_IoT using CyberValueCollator, our custom data collator, with a masking rate of 20%. After pretraining, the encoder is fine-tuned on Edge-IIoTset through a dual-head multitask classification setup: a binary head that classifies traffic as attack or benign, and a multiclass head that identifies the specific attack category. Both heads operate on the hidden-state representation of the <s> token, extracted from the final encoder layer, with a dropout rate of 0.1 applied before each classification head as a regularization mechanism. The final training objective is a weighted linear combination of the two task-specific losses:

(1) $$L=0.3\cdot L_{\mathrm{binary}}+0.7\cdot L_{\mathrm{multiclass}}$$

where $L_{\mathrm{binary}}$ is the binary head’s cross-entropy loss and $L_{\mathrm{multi}}$ is the multiclass head’s loss. The unequal weighting reflects the greater difficulty of the multiclass objective. Since no class balancing was performed on the data itself, we additionally applied inverse-frequency class weights:

(2) $$w_{c}=\frac{N_{\mathrm{total}}}{N_{c}}$$

where $N_{\mathrm{total}}$ is the total number of samples and $N_{c}$ is the number of samples in class *c*. The resulting weights are normalized by their mean, so the average weight across classes is 1, and the weighted loss remains on the same scale as an unweighted loss.

#### 3.4.1. Cyber Tokenizer

A crucial component of our CyberRoBERTa framework is the tokenizer, which acts as the translator between network-flow records and the encoder model. Conventional tokenizers, such as WordPiece (used by BERT) and Byte Pair Encoding (used by RoBERTa), are trained on general text corpora such as Wikipedia pages, books, and articles. These tokenizers are built for natural language, where subword segmentation is optimized for linguistic patterns rather than for structured, numerically dense data such as IoT network traffic. A feature name such as bidirectional_stddev_piat_ms, tokenized without domain adaptation, would be split into multiple small subword pieces, none of which individually correspond to a meaningful unit in the network-flow schema.

We trained the tokenizer from scratch on the pretraining corpus using the HuggingFace Unigram implementation, with a target vocabulary of 8000 tokens plus 57 locked feature-name tokens, injected as atomic units so that the trainer treats each one as a single, indivisible unit rather than fragmenting it during vocabulary pruning. This preserves the semantic identity of each feature name across all records, regardless of the surrounding numerical context. NFKC Unicode normalization was applied during pre-tokenization to standardize character representations while preserving characters critical to the serialization format, namely, the equals sign (=), the period (.), and the underscore (_). A whitespace-split pretokenizer respects the key = value boundaries of each flow record, and RoBERTa-style post-processing (via TemplateProcessing) wraps each encoded sequence with beginning-of-sequence (<s>) and end-of-sequence (</s>) tokens.To further characterize this tokenizer’s contribution relative to sequence-length effects, tokenizer design itself, and pretraining scale, we conducted three additional controlled comparisons, described in Section 4.3.

#### 3.4.2. CyberValueCollator

To train CyberRoBERTa effectively on structured network traffic, we designed a custom data collator, CyberValueCollator, tailored to the header_name = value format of our serialized data, where each value is a numerical measurement (e.g., packet length, flow duration, byte count). This structure calls for a corruption strategy that targets numerical values specifically, rather than arbitrary subword tokens as in standard masked language modeling.

CyberValueCollator corrupts only the numerical values in each sequence, leaving feature-name tokens intact, following a 10-10-80 scheme: 10% of value tokens are left unchanged, 10% are replaced with a random numerical value sampled from the training vocabulary, and the remaining 80% are replaced with the [MASK] token, at an overall masking rate of 20%. Feature-name tokens are excluded from masking entirely: since these names remain constant regardless of network environment or attack type, masking them would add noise to the pretraining gradient without providing a meaningful signal about traffic behavior. Protected tokens are identified directly from the locked feature-token list rather than inferred from vocabulary patterns, and this protection is verified programmatically before training begins.

Further experiments were conducted to assess whether this global-random substitution strategy is optimal, pretraining two additional encoders identical in every respect to the 20%-masking configuration except for the mechanism governing the 10% random-replacement slice: a zero-replacement variant, in which corrupted value tokens are substituted with a fixed placeholder rather than a sampled value, and an intra-feature swap noise variant, in which corrupted value tokens are substituted only with other real values belonging to the same feature, drawn from a pool of that feature’s observed values precomputed across the training corpus. This design is motivated by prior work on tabular representation learning, which finds that corruption strategies constrained to respect a table’s column structure yield stronger learned representations than unconstrained vocabulary-wide substitution [29]. We fine-tune each pretrained encoder using the identical procedure described in Section 3.4.5; results are reported in Section 4.1.2.

#### 3.4.3. Classification Heads

After pretraining, CyberRoBERTa is fine-tuned for intrusion detection through a dual-head multitask classification setup. Two classification heads operate in parallel on the encoder’s output: a binary head that classifies traffic as attack or benign, and a multiclass head that identifies the specific attack category. Both heads operate on the same hidden-state representation, extracted from the <s> token, the first token of every RoBERTa sequence, which aggregates information across all positions via bidirectional self-attention. A dropout layer (rate 0.1) is applied to this hidden state (drawn directly from the final encoder layer via last_hidden_state[:, 0]) before each classification head, serving as regularization against overfitting during fine-tuning.

#### 3.4.4. Pretraining Configuration

CyberRoBERTa was pretrained from scratch on the CICIoT2023 and TON_IoT benign corpus. Sequence-length analysis of the full serialized corpus showed a mean length of 254.45 tokens, with the 95th percentile at 301 tokens and a maximum of 355 tokens; based on this distribution, a fixed maximum sequence length of 400 tokens was chosen, comfortably covering the full range of observed flow lengths without introducing excessive padding overhead. The learning rate was set to $5\times 10^{-5}$, decayed linearly over training and preceded by a 500-step warmup, reflecting the need for a stable initialization when training a transformer encoder entirely from scratch rather than fine-tuning existing weights. To balance GPU memory constraints against training stability, an effective batch size of 64 was achieved through gradient accumulation: each device processed a batch of 16 samples at a time, accumulated over four steps before each optimizer update. A weight decay of 0.01 was applied for regularization. Masking followed the CyberValueCollator scheme described above, corrupting 20% of behavioral value tokens while leaving schema tokens untouched. Training ran for a maximum of 5 epochs, with validation loss evaluated every 100 steps and model checkpoints saved every 500 steps, retaining the two most recent checkpoints. Early stopping was applied when validation loss failed to improve over 3 consecutive evaluation checkpoints, preventing overfitting during the extended pretraining process. Mixed-precision (FP16) training was enabled throughout to reduce memory usage and accelerate training on the available hardware. The pretraining corpus was split 90%/10% into training and validation sets.

#### 3.4.5. Fine-Tuning Configuration

The pretrained CyberRoBERTa encoder was loaded alongside the dual-head multitask classification architecture described above and fine-tuned on Edge-IIoTset using a 90/10 train/test split with a fixed random seed. Fine-tuning used a maximum sequence length of 256 tokens, shorter than the 400-token length used during pretraining, reflecting the representation needs of individual labeled flows rather than the fuller corpus-level distribution considered during pretraining. A lower learning rate of $1\times 10^{-5}$ was used, relative to the $5\times 10^{-5}$ used during pretraining, since fine-tuning adapts an already-trained encoder and therefore requires smaller parameter updates to avoid disrupting the representations learned during pretraining. Fine-tuning ran for up to 6 epochs, with the class-weighted combined loss described above (Equations (1) and (2)) applied throughout to counteract label imbalance across attack categories. Early stopping was based on macro-averaged F1-score rather than validation loss or accuracy, since macro F1 weights every attack category equally regardless of sample count, making it a more reliable indicator of balanced classification performance across both frequent and underrepresented classes.

#### 3.4.6. Evaluation Metrics

Model evaluation is reported separately for the binary and multiclass classification heads. For the binary head, we report the accuracy, precision, recall, F1-score, and the ROC curve, with F1-score and AUC serving as the primary summary metrics; F1-score captures the combined cost of missed attacks and false alarms without being inflated by the true negatives typical of an imbalanced dataset such as ours. The same metrics are applied to the multiclass head, with the addition of a confusion matrix.

#### 3.4.7. Hardware and Software Environment

All experiments, dataset preprocessing, tokenizer training, pretraining, the masking-rate ablation, fine-tuning and evaluation, the tokenizer-scale isolation experiments (Section 4.3.2), the classical machine learning baseline’s training and accuracy evaluation (Section 4.5), the class-imbalance interventions (Section 4.6), and the value-replacement ablation (Section 4.1.2), were conducted on a single NVIDIA Tesla T4 GPU with 16 GB of VRAM via Google Colab. CPU-only inference latency measurements (Section 4.8) were obtained on a CPU-only Google Colab runtime to approximate constrained-hardware inference conditions. The classical machine learning baseline’s inference latency (also reported in Section 4.5) was likewise measured on this CPU-only runtime, alongside CyberRoBERTa’s, to ensure a fair, matched-hardware latency comparison between the two. Software dependencies included Python 3.13.15, PyTorch 2.11.0, the HuggingFace transformers (5.18.0), tokenizers (0.23.2), and datasets (4.8.5) libraries, scikit-learn (1.6.1) for evaluation metrics and the XGBoost baseline, xgboost (3.4.1) for the classical machine learning comparison, and imbalanced-learn (0.14.2) for SMOTE-based oversampling.

## 4. Results

CyberRoBERTa is evaluated on the Edge-IIoTset dataset, comprising 2639 labeled IoT network flow records across 12 classes (11 attack categories plus normal traffic). The model was evaluated across multiple masking-rate configurations before being assessed on the downstream intrusion detection task.

### 4.1. CyberValueCollator Configuration Ablations

CyberValueCollator’s behavior is governed by two independent design choices: how much of a flow’s numerical content is corrupted during pretraining, and how corrupted values are replaced once selected. We evaluate both dimensions in turn.

#### 4.1.1. Masking Ablation

The selected encoder model was trained with the domain-specific tokenizer using the CyberValueCollator. We tested our CyberValueCollator on four masking rates, 15%, 20%, 25%, and 30%, with an additional baseline experiment using the standard HuggingFace data collator at 15%, to determine whether a higher masking rate improves downstream attack detection, as shown in Table 4. The 20% CyberValueCollator configuration achieved the highest macro-averaged F1 of 0.8422, ahead of the next-best configurations, 25% CyberValueCollator (0.8362) and the 15% Standard baseline (0.8361). At the per-class level, the picture is more nuanced: the 20% configuration achieved the best results on HTTP_Flood, Ransomware, and XSS, and tied with the 25% configuration on Scanning Vulnerability. The 15% CyberValueCollator configuration performed best on Backdoor and MITM, while the 15% Standard baseline performed best on Normal and tied with the 25% configuration on Password. On Port_Scanning, the 20% configuration was in fact the only one of the five that did not reach the shared ceiling score achieved by the other four. Nevertheless, the 20% configuration achieved the best or tied-best F1-score across seven of the twelve attack categories and produced the highest macro-averaged F1-score among all five configurations. The 20% model was therefore selected for downstream evaluation.

#### 4.1.2. Value-Replacement Strategy in CyberValueCollator

Table 5 reports per-class F1 for the three value-replacement configurations: global-random (published), intra-feature swap, and zero-replacement Macro F1 across the three configurations ranged from 0.8422 to 0.8473, with zero-replacement scoring marginally higher than the other two. At the per-class level, both zero-replacement and intra-feature swap improved MITM detection from 0.4138 under global-random substitution to 0.6429, an identical result obtained from two independently pretrained encoders. However, both approaches left Backdoor and Ransomware essentially unchanged or slightly degraded relative to the global-random configuration.

### 4.2. Pretraining Convergence

**Figure 3:**
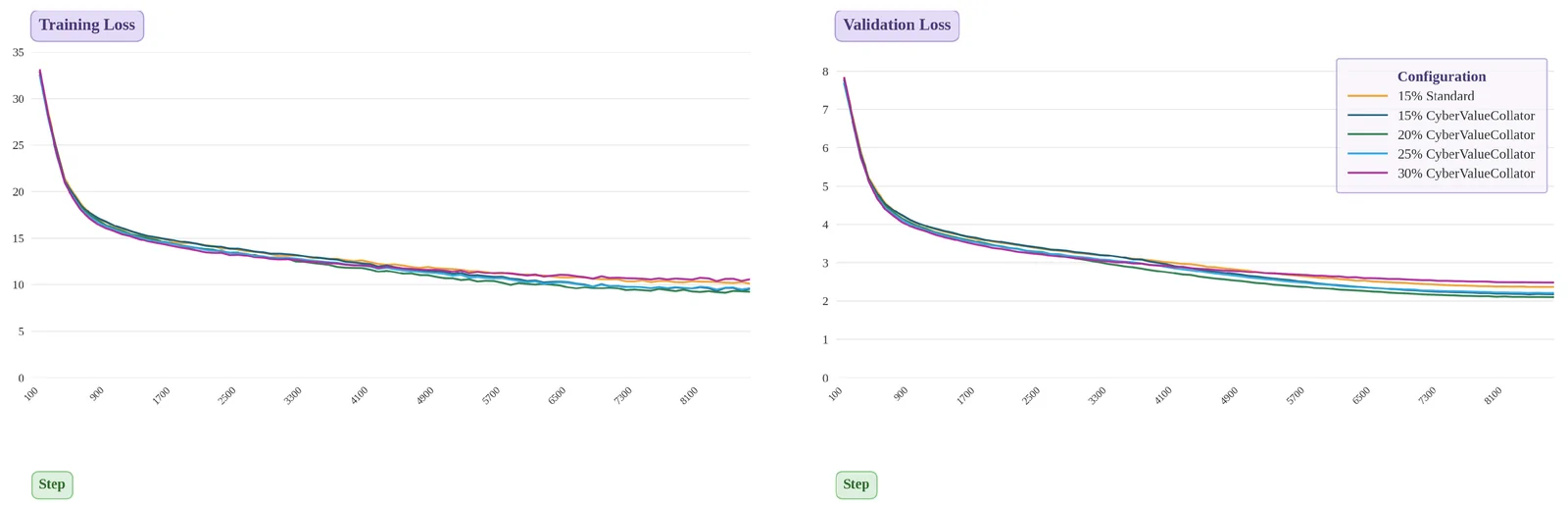
Training and validation loss during pretraining, across all five masking configurations.

CyberRoBERTa’s encoder was pretrained for five epochs (8790 steps total) under each of the five masking configurations evaluated in the masking-rate ablation (Section 4.1.1), using identical architecture and optimization settings across all five runs. Training and validation loss are logged every 100 steps throughout pretraining; Figure 3 shows the complete validation and training loss curves for all five configurations. For the 20% CyberValueCollator configuration, ultimately selected for the final model, training loss decreased steadily throughout pretraining, from 32.49 at step 100 to 9.22 by step 8700, a reduction of approximately 72%. Validation loss followed a corresponding trend, decreasing from 7.67 at step 100 to 2.10 by step 8700, with no divergence between training and validation loss observed at any point during training, indicating that the model did not overfit to the pretraining corpus. Validation loss showed diminishing returns over the final portion of training: from step 7900 onward, validation loss for this configuration remained within a narrow band of approximately 2.10 to 2.13, indicating that the encoder had largely converged on the pretraining objective by this point. This plateau is consistent with the early stopping criterion described in Section 3.4.4 under which training terminates once validation loss fails to improve meaningfully over consecutive evaluation checkpoints.

Comparing across all five configurations reveals a more nuanced convergence pattern than final loss values alone suggest. Over approximately the first 2000 steps, the 30% CyberValueCollator configuration converged fastest, achieving the lowest validation loss of any configuration at steps 500 through 2000. From approximately step 3000 onward, however, this ordering reverses: the 20% CyberValueCollator configuration achieves the lowest validation loss of all five configurations at every subsequent logged checkpoint, ending at a final validation loss of 2.10, compared to 2.18 for 15% CyberValueCollator, 2.21 for 25% CyberValueCollator, 2.37 for 15% Standard, and 2.48 for 30% CyberValueCollator. This pattern suggests that higher masking rates provide a stronger early learning signal, since a larger proportion of corrupted tokens per batch yields more gradient signal per step, but this advantage does not persist: at 30% masking, the corruption rate may be too aggressive to support continued refinement over longer training, while the 20% configuration reaches a better long-run optimum despite slower initial progress. Notably, the 20% configuration that achieves the lowest pretraining validation loss is the same configuration independently selected based on downstream fine-tuning performance (Table 4), providing corroborating evidence, from an upstream, task-agnostic metric, for its selection.

Across all five configurations, no divergence between training and validation loss was observed at any point during pretraining, indicating that overfitting to the pretraining corpus was not a concern for any of the evaluated masking rates.

### 4.3. Tokenizer Characterization and Scale Isolation

We first characterize the domain-specific tokenizer’s mechanical properties relative to a generic alternative, then investigate whether the resulting differences reflect tokenizer design itself or confounding factors such as sequence truncation and pretraining scale.

#### 4.3.1. Tokenizer Characterization

To assess whether domain-specific tokenization provides a meaningful advantage over generic, off-the-shelf tokenization for IoT network traffic, we compared our Unigram-based tokenizer against the pretrained tokenizer distributed with roberta-base, applied to a random sample of 5000 flows drawn from the same pretraining corpus (fixed seed), without modification or retraining. At the level of individual feature names, the domain-specific tokenizer preserved every tested feature name as a single, intact token, while the generic tokenizer fragmented every tested feature name into 6 to 12 unrelated subword pieces; for example, bidirectional_stddev_piat_ms was split into 12 fragments, none of which individually correspond to a meaningful unit in the network-flow schema. This difference compounds at the corpus level. Flows tokenized with the generic tokenizer required a mean of 928.87 tokens, compared to 253.58 tokens for the domain-specific tokenizer, an increase of approximately 3.7 times. At the 95th percentile, generic tokenization produced sequences of 958 tokens, exceeding roberta-base’s own 512-token position limit; the domain-specific tokenizer’s 95th-percentile sequence length of 301 tokens remained well within the 400-token maximum used during pretraining. Vocabulary utilization also differed substantially: the generic tokenizer used only 2.87% of its 50,265-token vocabulary on our corpus (1442 tokens), compared to 95.60% utilization for the domain-specific tokenizer’s 8000-token vocabulary (7648 tokens). Both tokenizers achieved a 0% unknown-token rate and 100% roundtrip fidelity, though the former is a structural property of roberta-base’s byte-level encoding rather than evidence of domain fit. Table 6 summarizes these results.

#### 4.3.2. Isolating Tokenizer Design, Truncation, and Pretraining Scale

Table 7 reports macro F1 across the four tokenizer, truncation, and pretraining-scale conditions.

The truncated and extended roberta-base variants performed comparably, with the truncated variant scoring slightly higher (0.8487 vs. 0.8468). A second comparison isolates tokenizer design directly: holding pretraining corpus and architecture fixed and varying only the tokenizer, CyberRoBERTa (0.8422) outperforms a generic-tokenizer model trained under identical conditions (0.8366), despite that model having 2.3 times more parameters. This advantage disappears, however, once pretraining scale is allowed to vary: both roberta-base variants outperform CyberRoBERTa (0.8487 and 0.8468 vs. 0.8422).

### 4.4. Downstream Performance

The binary classification head of the selected 20% configuration achieved an overall F1-score of 96%, with 96% F1 on normal traffic and 97% on attack traffic, demonstrating strong, balanced performance across both classes. The attack class scored 94% precision and 100% recall, while the normal class achieved 100% precision and 92% recall. These results illustrate the model’s ability to maintain high attack sensitivity (a recall of 100%) without sacrificing precision on normal traffic. Full results are presented in Table 8.

The multiclass head achieved an overall F1-score of 89.54%, a macro-average F1 of 84.22%, and a weighted-average F1 of 91.26%. Table 9 shows that while the encoder model is strong at detecting high-volume attacks such as TCP SYN flood, UDP flood, and HTTP flood, it struggles with more ambiguous classes such as Backdoor, MITM, and Ransomware. Further examination of the results reveals three distinct detection patterns.

First, the perfect-detection group comprises attack classes that achieved an F1-score of 100% with zero misclassifications. TCP SYN flood (F1: 1.0000), UDP flood (F1: 1.0000), and SQL injection (F1: 1.0000) fall under this category. These attack types generate abnormal network behaviors; for instance, TCP SYN flood and UDP flood generate one-directional, high-volume traffic with irregular arrival times. SQL injection, by contrast, cannot be observed through explicit protocol flags, yet it shows a distinct directional asymmetry in byte distribution. A relatively consistent inter-arrival time combined with large packet sizes further suggests automated request generation and structured payload delivery, indicative of complex, repeated submissions.

Strong-detection classes fall within the 78.12%–99.33% F1-score range and include HTTP Flood (F1: 0.9933), Password (F1: 0.9855), Port Scanning (F1: 0.9670), Scanning Vulnerability (F1: 0.9635), Normal traffic (F1: 0.9581), and XSS (F1: 0.7812). HTTP Flood is distinguishable through heavy, sustained, repetitive traffic. Password attacks, a form of brute-force attack, are detected through many short sessions with repeated authentication attempts. Port Scanning and Scanning Vulnerability are two closely related attack types that share low packet counts and high connection counts; both are detected with F1-scores of 96.70% and 96.35%, respectively. Finally, XSS achieves an overall F1-score of 78.12%, with a precision of 100.00% but a recall of only 64.10%, indicating that while the model correctly identifies XSS when flagged, it misses 35.90% of actual XSS flows.

Challenging-detection classes fall in the F1 range of 39.56%–64.78%: Backdoor (F1: 0.6478), MITM (F1: 0.4138), and Ransomware (F1: 0.3956) are the three most difficult classes to detect. Backdoor shows a moderately balanced precision-recall profile (74.07% precision, 57.55% recall), reflecting that the model has partially learned the Backdoor behavioral pattern but cannot reliably separate it from low-volume attack types, specifically Ransomware. Backdoor attacks demonstrate irregular, low-frequency sessions whose inter-arrival-time and packet-size statistics overlap with Ransomware, making the two fundamentally difficult to distinguish. MITM demonstrates a sharp precision-recall imbalance: it is correct 85.71% of the time it is predicted, but it identifies only 27.27% of actual MITM flows, with the remainder misclassified. This is consistent with MITM’s known tendency to mimic normal session patterns to evade detection; the severe underrepresentation of MITM in the training data (187 samples) may also indicate the model had insufficient exposure to learn the attack pattern thoroughly. Ransomware shows the inverse pattern to MITM, with low precision (26.97%) but strong recall (74.17%). These results indicate that the model captures 74.17% of ransomware flows, yet many flows from other attack categories are misclassified as Ransomware, producing a large false-positive burden for this class. This reflects the behavioral similarities between ransomware, normal traffic, and backdoor activity, all of which generate low-frequency encrypted sessions with irregular inter-arrival times. While the ransomware recall reached 74.17% and the backdoor recall reached 57.55%, the model evidently cannot fully distinguish between the two.

**Figure 4:**
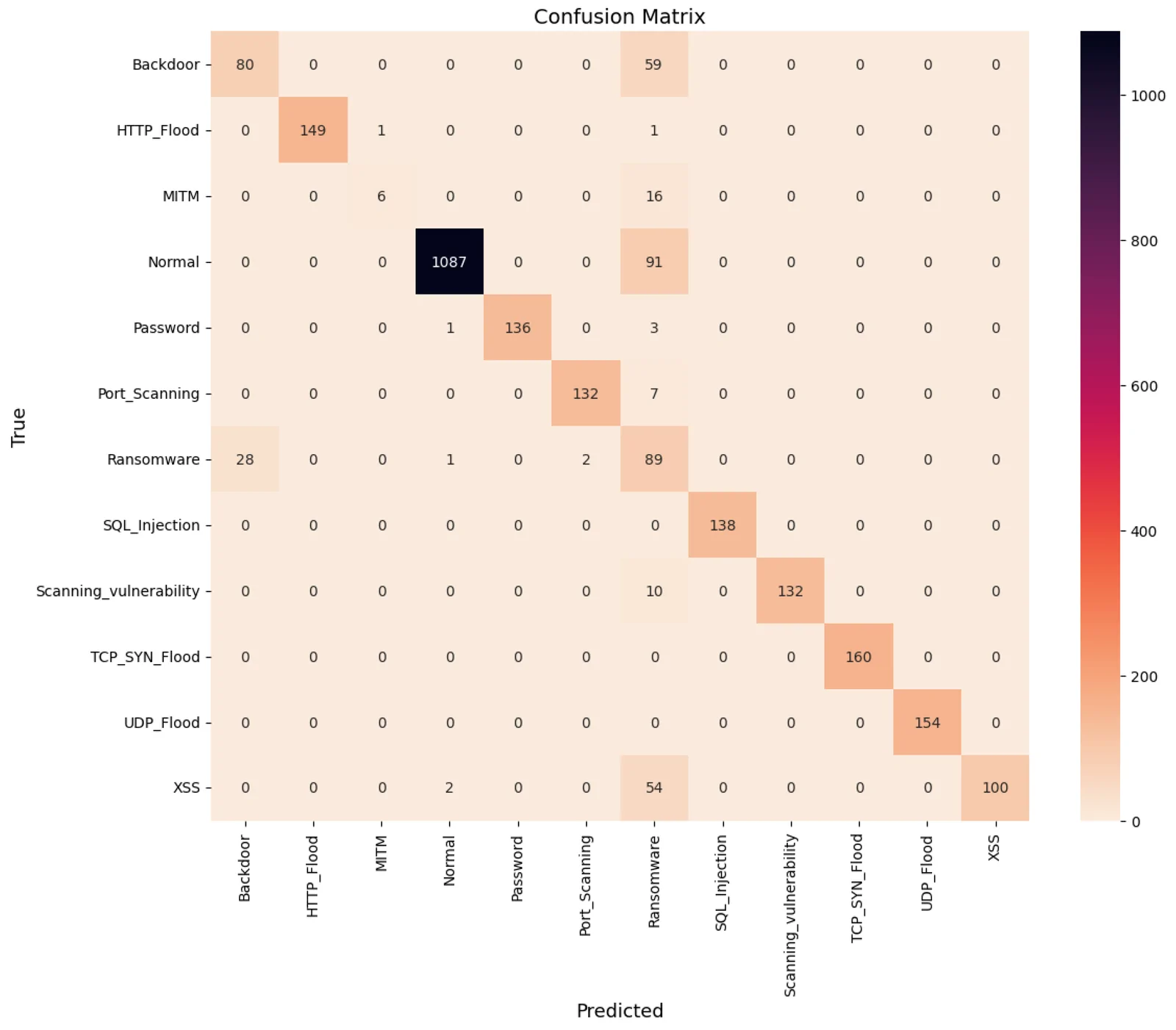
Confusion matrix, multiclass classification head.

Figure 4 illustrates the confusion matrix for the multiclass classification head, evaluated on 2639 held-out test flows. The matrix reveals three notable patterns. First, the Ransomware column acts as a dominant misclassification sink: it absorbs 59 misclassified Backdoor flows, 91 Normal flows, 16 MITM flows, 54 XSS flows, 10 Scanning Vulnerability flows, 7 Port Scanning flows, 3 Password flows, and 1 HTTP Flood flow, totaling 241 misclassified flows. Ransomware itself correctly identifies only 89 of its 120 true instances, with the remaining 31 distributed across Backdoor (28), Port Scanning (2), and Normal (1). The Normal class correctly identifies 1087 of its 1178 instances, with 91 flows leaking into the Ransomware column, explaining that class’s low precision of 26.97%. Second, MITM represents a clear recall failure: only six samples were correctly identified, and 16 were misclassified as Ransomware, explaining MITM’s high precision, since on the rare occasions the model flagged MITM, it was almost always correct. Third, XSS was correctly classified in 100 of its 156 instances, though 54 flows leak into the Ransomware column and 2 into Normal traffic.

**Figure 5:**
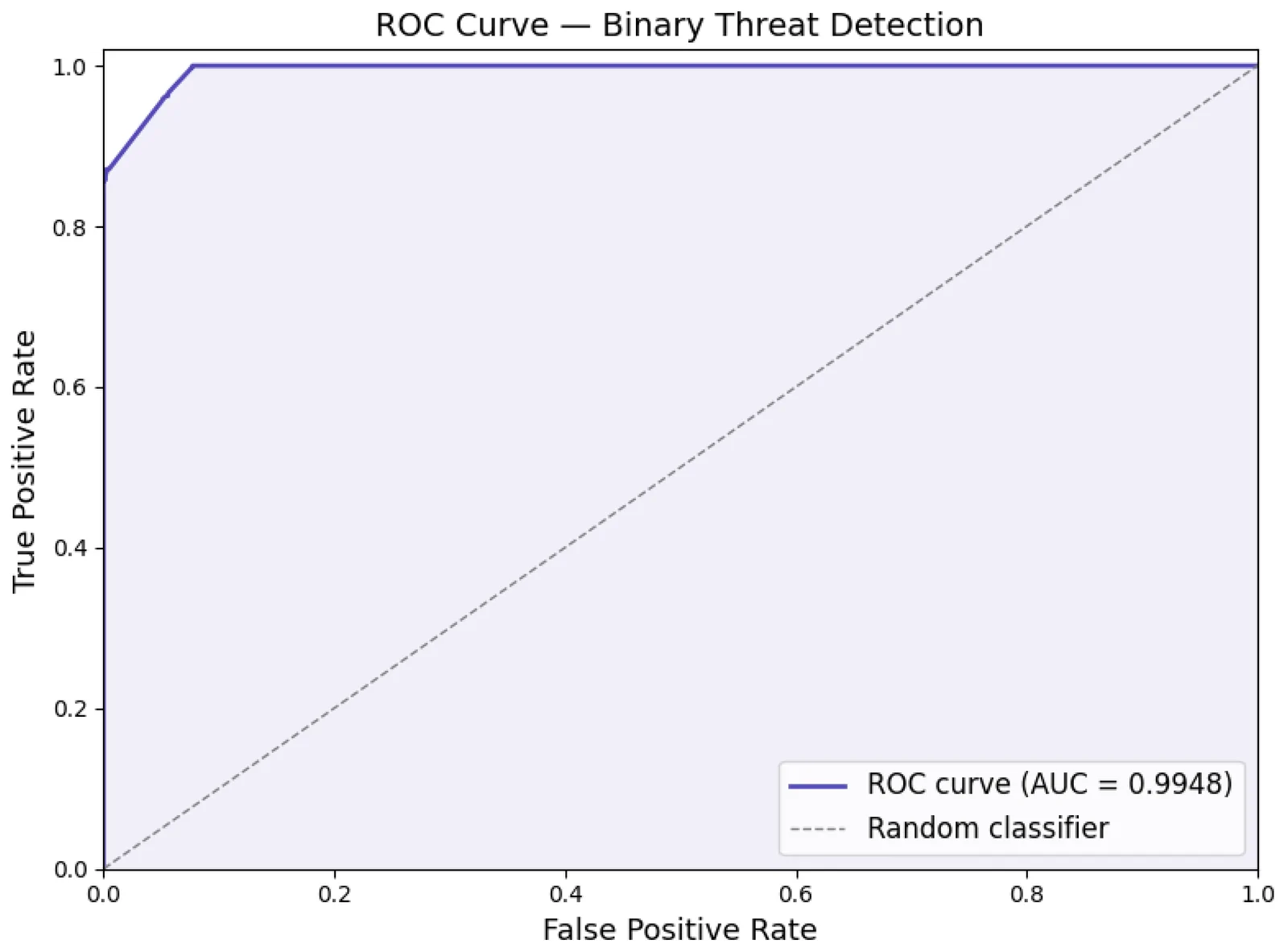
ROC curve, binary classification head.

Figure 5 presents the receiver operating characteristic (ROC) curve for the binary classification head, plotting the true positive rate against the false positive rate across all possible classification thresholds. The curve rises sharply toward the top-left corner of the plot, reaching a true positive rate near 1.0 while the false positive rate remains close to 0, and yields an area under the curve (AUC) of 0.9948. This near-perfect AUC indicates that the binary head separates attack traffic from normal traffic with high confidence across essentially the full range of decision thresholds, rather than only at the single default threshold used to compute the precision, recall, and F1-scores reported in Table 8. In practical terms, this means that the strong recall and precision already observed are not an artifact of a fortunately chosen threshold: even fairly conservative or aggressive threshold settings would preserve most of the model’s discriminative power, which is a desirable property for an intrusion detection system that may need to be tuned toward higher sensitivity or higher specificity depending on deployment context.

### 4.5. Comparison Against a Classical Machine Learning Baseline

To evaluate whether CyberRoBERTa’s architectural complexity is necessary for this task, we compared it against XGBoost, a gradient-boosted tree ensemble trained on the identical 57 retained features used throughout this study. Both models were trained and evaluated on the same fine-tuning corpus and 90/10 split, with equivalent inverse-frequency class weighting (Equation (2)) applied to both, isolating model architecture as the variable under comparison. XGBoost’s full results are reported in Table 10 and Table 11.

At the binary level, the two models are close but not identical. XGBoost holds a small, consistent edge across every metric, namely, higher Normal recall (0.94 vs. 0.92), higher Attack precision (0.95 vs. 0.94), and higher ROC AUC (0.9956 vs. 0.9948). None of these individual gaps is large; however, their consistency in the same direction across precision, recall, and AUC indicates a genuine, if modest, binary-classification advantage for XGBoost. At the multiclass level, several classes are effectively tied between the two models. SQL Injection, TCP SYN Flood, and UDP Flood both score 1.00 F1 for CyberRoBERTa and XGBoost alike. HTTP Flood is nearly identical as well (0.99 vs. 0.99), as is Password (0.99 vs. 0.99) and Port Scanning (0.97 vs. 0.97).

A small number of classes differ by 1 to 2 points. Normal favors XGBoost slightly (0.96 vs. 0.97), and Scanning Vulnerability favors CyberRoBERTa slightly (0.96 vs. 0.94).

Three classes differ by a more noticeable 4 to 6 points. Backdoor favors CyberRoBERTa (0.65 vs. 0.59). Ransomware favors XGBoost (0.40 vs. 0.46). XSS favors XGBoost (0.78 vs. 0.82).

MITM stands apart from every other class by a wide margin. CyberRoBERTa’s F1 exceeds XGBoost’s by 22 points (0.41 vs. 0.19), several times larger than any other class’s difference, and the two models arrive at this gap through opposite error patterns: XGBoost predicts MITM far more often but is correct only 11% of the time it does, while CyberRoBERTa predicts it less often but is correct far more consistently when it does.

On computational cost, the two models differ sharply. XGBoost’s model size (4.19 MB in UBJ binary format, 4.66 MB in JSON) is approximately 9.3 times smaller than CyberRoBERTa’s (39.03 MB, FP32. Measured on identical CPU hardware, XGBoost’s mean single-flow inference latency (1.172 ms) was approximately 205 times lower than CyberRoBERTa’s (191 to 240 ms across repeated measurements; Section 4.8), with a corresponding throughput advantage (853.2 vs. 4.2 to 5.2 flows/second).

### 4.6. Class-Imbalance and Misclassification Interventions

Given MITM and Ransomware’s persistently low detection performance (Section 4.4), we evaluated three distinct interventions targeting these classes specifically: data-level resampling, loss-function reweighting, and classification-architecture restructuring. We applied each intervention independently, using the same pretrained CyberRoBERTa backbone and fine-tuning corpus to isolate its effect.

#### 4.6.1. SMOTE-Based Oversampling

We applied the Synthetic Minority Over-sampling Technique (SMOTE) to the training split, oversampling minority attack classes prior to fine-tuning, while retaining the existing inverse-frequency loss weighting (Equation (2)) unchanged. SMOTE generates synthetic minority-class examples by interpolating between existing real examples and their nearest neighbors in feature space, rather than duplicating them outright. Table 12 reports per-class F1 before and after this intervention.

Contrary to our expectation that oversampling would improve the detection of the most severely underrepresented classes, SMOTE provided no benefit and modestly reduced F1 for all three target classes; binary ROC AUC also declined slightly (0.9944 to 0.9939).

#### 4.6.2. Focal Loss

As a second intervention, we replaced the multiclass head’s standard weighted cross-entropy loss with focal loss [34], a loss function originally proposed for dense object detection that down-weights examples the model already classifies confidently and correctly, concentrating gradient signal on examples that remain difficult regardless of class membership. We retained the same inverse-frequency class weights described above as the loss’s α term, and set the focusing parameter γ=2, the standard default reported in the original formulation. Table 13 reports the resulting per-class F1.

Focal loss degraded MITM detection further than SMOTE. Training exhibited pronounced early-stage instability: in the earliest evaluation checkpoints, Backdoor, MITM, and Normal simultaneously showed precision and recall of exactly zero, indicating the model was not predicting these classes at all, before gradually recovering over subsequent checkpoints.

#### 4.6.3. Cascaded Classification Architecture

Unlike the two interventions above, which operate on the training data or loss function without altering model architecture, our third intervention restructured the classification pipeline itself. This was motivated by a specific diagnosis rather than a general imbalance-correction strategy: examination of the confusion matrix (Section 4.4) revealed that Ransomware’s low precision was driven substantially by real Normal-traffic flows being misclassified as Ransomware, accounting for 91 of 241 total misclassified flows attributed to that class. We hypothesized that removing Normal as a candidate label for the multiclass head specifically would reduce this source of error. We restructured the multiclass head to classify only among the 11 attack types, excluding Normal from its candidate label space entirely; under this cascaded design, the binary head alone determines Normal-versus-Attack status for a given flow, and the multiclass head is queried only to identify which specific attack type applies once a flow has already been classified as an attack. Table 14 reports the resulting per-class F1, compared against the original 12-way baseline.

Unlike SMOTE and focal loss, this intervention improved both target classes simultaneously: MITM improved by 0.127 and Ransomware by 0.056, the largest improvement for Ransomware across all interventions evaluated in this study. This result confirms that Normal-attack confusion was a genuine and directly addressable contributor to Ransomware’s difficulty. It also indicates a boundary on what this specific intervention can achieve: the improvement, while substantial, does not bring Ransomware or MITM to the performance level of well-separated classes, suggesting that attack-to-attack confusion—particularly between Backdoor and Ransomware, identified independently through feature-space analysis in Section 4.7—MITM remains an unresolved, structural source of difficulty that excluding Normal from the candidate label space does not address.

### 4.7. Model-Independent Verification of Feature-Space Overlap

The confusion-matrix analysis indicates that Backdoor, Ransomware, and MITM are frequently confused with one another and with Normal traffic. However, this pattern is observed through the lens of a single trained classifier, leaving open the possibility that it reflects a limitation specific to CyberRoBERTa’s training or representation rather than a genuine property of the underlying data. To verify this independently, we computed *k*-nearest-neighbor (k=10, Euclidean distance) label purity directly on the raw 57-feature representations, without training or querying any classifier. For each flow, we identified its 10 nearest neighbors in feature space and calculated the proportion sharing its true class label; a purity of 1.0 indicates a flow whose neighborhood is entirely its own class, while low purity indicates a flow in a feature-space region shared with other classes.

Table 15 reports purity by class. Backdoor, Ransomware, and MITM each show substantially lower purity (0.370, 0.438, and 0.224, respectively) than classes with near-perfect detection F1 throughout this study, such as SQL_Injection, TCP_SYN_Flood, and UDP_Flood, which retain purity at or near 1.000.

### 4.8. Inference Latency and Efficiency Analysis

To characterize where inference cost is concentrated within CyberRoBERTa’s pipeline, we measured per-component CPU latency across 200 real test flows: tokenization, the transformer encoder’s forward pass, and the dual classification heads, timed separately. Tokenization accounted for a mean of 2.89 ms (1.5% of total latency); the transformer encoder’s forward pass accounted for 188.26 ms (98.4%); and the two classification heads together accounted for 0.25 ms (0.1%). This confirms that inference cost is overwhelmingly attributable to the transformer encoder itself, with tokenization and classification contributing negligible overhead regardless of deployment context. Repeated measurement across sessions yielded end-to-end totals ranging from approximately 191 to 240 ms, reflecting normal variance in cloud CPU resource allocation rather than measurement error.

#### 4.8.1. FLOPs Analysis

Paralleling the empirical latency breakdown above, we computed theoretical FLOPs separately for each pipeline component. The transformer encoder requires 4.278 GFLOPs per forward pass (sequence length of 256 tokens); the two classification heads, each a single linear projection from the 320-dimensional pooled representation, require approximately 8960 FLOPs combined, roughly 477,000 times smaller than the encoder’s cost. This architectural estimate, obtained without running the model on any hardware, agrees with the empirical breakdown above: both independently attribute all inference cost to the transformer encoder, strengthening confidence that this reflects a genuine property of the model rather than an artifact of the specific hardware or software stack used.

#### 4.8.2. Model Footprint and Constrained-Hardware Projection

CyberRoBERTa’s complete model, comprising all 10,230,414 parameters, occupies 39.03 MB on disk in FP32 precision. Using the FLOPs figure above, we estimated inference latency on Raspberry Pi 4-class hardware, a device explicitly represented in the Edge-IIoTset testbed underlying this study’s fine-tuning corpus. Assuming a generic sustained throughput of 1–3 GFLOPS/second for unoptimized FP32 inference on such hardware, CyberRoBERTa’s 4.278 GFLOPs per forward pass implies a projected latency of approximately 2.1–4.3 s.

## 5. Discussion

### 5.1. Masking Rate and Collator Design

CyberValueCollator’s advantage over the standard 15% HuggingFace collator is not uniform across attack categories; however, it is most pronounced precisely where it is most consequential. The standard collator is the sole best-performing configuration only for the Normal class, and its remaining top scores (Password, Port_Scanning, SQL_Injection, TCP_SYN_Flood, UDP_Flood) are tied with two or more CyberValueCollator configurations on classes where nearly all configurations achieve near-ceiling performance regardless of masking scheme. By contrast, CyberValueCollator variants collectively achieve the best or tied-best performance on the three most difficult classes in the benchmark, namely, Backdoor, MITM, and Ransomware, over which the standard collator never attains the best individual result. This pattern is corroborated at the aggregate level, though only for the 20% configuration: 20% CyberValueCollator attains a macro-F1 of 0.8422, exceeding the 15% standard baseline (0.8361). The 25% CyberValueCollator configuration (0.8362) and the 15% standard baseline (0.8361), however, are indistinguishable, while 15% CyberValueCollator (0.8305) trails the baseline modestly. Moreover, the 30% CyberValueCollator configuration (0.8276) exhibited a clear decline in performance relative to the other four masking rates, motivating the decision not to evaluate masking rates beyond this point. These findings indicate that the separation of schema tokens from behavioral tokens confers a measurable aggregate benefit specifically at the 20% masking rate, rather than uniformly across CyberValueCollator configurations; separating schema tokens from behavioral tokens, in other words, only yields a measurable benefit once a sufficient degree of corruption, at least 20%, is concentrated entirely on behavioral values. Restricting the comparison to CyberValueCollator configurations alone, the rise from 15% to a peak at 20% and the subsequent decline at 25% and 30% masking suggests a tradeoff between bias and signal rather than a simple “more masking is better” relationship: light masking under-corrupts the behavioral tokens the model needs to reconstruct, while masking beyond roughly a quarter of value tokens strips out enough surrounding numerical context that the reconstruction task becomes under-determined, and the 20% setting appears to sit close to the point where corruption is aggressive enough to force genuine inference without collapsing a flow’s behavioral coherence. Moreover, this pattern shows that masking rate is not an incidental hyperparameter but one that materially governs the collator’s downstream effectiveness, with a margin of about 1.5 percentage points separating the best- and worst-performing configurations.

Beyond masking rate, the mechanism governing how corrupted values are replaced also proves consequential, though selectively so. Feature-constrained substitution, whether via a fixed placeholder (zero-replacement) or via values drawn only from the same feature’s own distribution (intra-feature swap noise), introduces less extraneous statistical noise into the pretraining signal than CyberValueCollator’s default global-random substitution, which can replace a value with one drawn from an entirely unrelated feature. Both alternatives improved MITM F1 substantially, from 0.4138 to 0.6429 (Section 4.1.2), an identical result obtained from two independently pretrained encoders. For a severely data-scarce class such as MITM, this cleaner corruption signal plausibly allows the model to learn a more reliable representation from the limited real examples available, since less of the pretraining signal is spent reconstructing implausible, cross-feature substitutions.

This result may appear, at first glance, to be in tension with the feature-space purity analysis (Section 4.7), where MITM registered the lowest purity of the three difficult classes (0.224), lower than both Backdoor (0.370) and Ransomware (0.438), yet MITM is the class that improved most under this intervention, while Ransomware, with comparatively higher purity, improved little and Backdoor did not improve at all. We note that purity and sample scarcity are distinct, independently measured properties, and MITM is an extreme case on both axes simultaneously: it has not only the lowest purity but also, by a wide margin, the fewest real training examples, whereas Backdoor and Ransomware have moderate purity and comparatively more real examples available. We report this pattern: the one class improved by this intervention is also the one class that is an outlier on both purity and scarcity simultaneously.

### 5.2. Tokenizer Design, Truncation, and Pretraining Scale

The tokenizer-scale isolation results (Section 4.3.2) clarify what drives the F1-score differences observed across tokenizer and pretraining conditions. That the truncated and extended roberta-base variants perform comparably indicates that truncation avoidance is not what accounts for the generic tokenizer’s disadvantage relative to CyberRoBERTa in Section 4.3.2. In contrast, isolating the tokenizer’s contribution shows that when pretraining corpus and architecture are held fixed, CyberRoBERTa’s domain-specific tokenizer outperforms a generic tokenizer trained under identical conditions, despite the generic-tokenizer model having 2.3 times more parameters. Nevertheless, this advantage disappears once pretraining scale is allowed to vary: both roberta-base variants, benefiting from several gigabytes of general-domain pretraining rather than the ∼197MB IoT-specific corpus available for this study, outperform CyberRoBERTa.

Domain-specific tokenization is not advantageous independent of encoder scale in an unconditional sense: sufficient pretraining-data volume can outweigh tokenizer design entirely, as it does here. The evidence instead supports a narrower, fully controlled claim: Given equal pretraining conditions, the realistic scenario for any domain-specific model trained from scratch on a corpus of the size available in this study rather than one benefiting from web-scale external pretraining, domain-specific tokenization provides a genuine, measurable advantage over generic tokenization, independent of truncation effects.

### 5.3. Machine Learning Comparison

At the binary level, both models achieve strong, near-ceiling detection of Attack versus Normal traffic, with XGBoost holding a small, consistent edge across every metric, precision, recall, F1, and AUC alike.

On the multiclass level, CyberRoBERTa’s MITM precision-oriented profile (0.85 precision, 0.27 recall, F1 0.41) contrasts sharply with XGBoost’s recall-oriented profile (0.11 precision, 0.53 recall, F1 0.19): CyberRoBERTa’s F1 is more than double XGBoost’s, and the two models fail in opposite ways. XGBoost flags MITM far more often but is correct only 11% of the time it does so, whereas CyberRoBERTa is more conservative but considerably more trustworthy when it flags MITM. On Ransomware, the pattern reverses: XGBoost achieves a meaningfully higher F1 (0.46 vs. 0.39), driven by higher precision (0.37 vs. 0.26) at the cost of lower recall (0.60 vs. 0.74). In other words, XGBoost produces fewer Ransomware false positives, but it misses more true Ransomware flows than CyberRoBERTa. On Backdoor, CyberRoBERTa achieves a modest F1 advantage over XGBoost (0.64 vs.0.59). This advantage is driven primarily by precision: CyberRoBERTa correctly identifies Backdoor 74% of the time it predicts the class, compared to XGBoost’s 65%, while the two models’ recall is closer (57% vs. 53%). Nevertheless, both CyberRoBERTa and XGBoost achieve near-identical, near-ceiling detection on the same set of classes: SQL Injection, TCP SYN Flood, UDP Flood, HTTP Flood, and Password all score above 0.98 F1 for both models. Port Scanning and Scanning Vulnerability follow closely, though with a small divergence between the two models: CyberRoBERTa holds the advantage on Scanning Vulnerability by roughly 2 points (0.96 vs. 0.94), while XGBoost holds a much smaller advantage on Port Scanning, under one point (0.96 vs. 0.97). XSS, by contrast, scores noticeably lower on both (0.78 to 0.82 F1), with XGBoost ahead by roughly 3.4 points, and we discuss it alongside Backdoor in the moderate tier below. MITM, however, has shown an interesting trajectory throughout these experiments. XGBoost achieved only 0.19 F1 on MITM, while CyberRoBERTa’s 20% CyberValueCollator configuration more than doubled this at 0.41, and a further experiment, the value-replacement ablation, raised this still further to 0.64.

Nevertheless, these results are Consistent with a broader, well-documented pattern in the tabular-data literature, where tree-based ensembles such as XGBoost are frequently reported to match or exceed transformer-based architectures on structured, feature-engineered data [35], XGBoost performs competitively with CyberRoBERTa across most of this benchmark, and it holds a modest advantage in aggregate binary classification, on Ransomware, and on inference cost. CyberRoBERTa’s distinguishing result is narrower and more specific: on MITM, the single most data-scarce and behaviorally camouflaged class in this study, its F1 is more than double XGBoost’s, with XGBoost’s low precision indicating it identifies MITM candidates largely by over-flagging rather than genuine discrimination. We note that CyberRoBERTa and XGBoost perform comparably across both weighted and macro-averaged F1: weighted F1 is nearly tied (0.9126 vs. 0.9147), while macro F1 shows a small advantage for CyberRoBERTa (0.84 vs. 0.83). This is consistent with the same underlying pattern: weighted F1 is influenced more heavily by performance on the largest class, Normal, where XGBoost holds a marginal edge (0.97 vs. 0.96 F1); macro F1 weights every class equally regardless of size, so CyberRoBERTa’s substantial MITM advantage (0.41 vs. 0.19, on only 19 test flows) contributes proportionally more to this metric.

### 5.4. Feature-Space Overlap and Attack Mechanism

This model-independent geometric analysis directly corroborates the confusion-matrix-based diagnosis above: Backdoor, Ransomware, and MITM occupy substantially overlapping regions of the feature space, a structural property of the available 57 features that no fine-tuning-stage intervention, including those evaluated in Section 4.6, can be expected to fully resolve, since no classifier can separate examples with near-identical feature representations but differing labels.

A closer examination of the attack mechanism, rather than the protocol layer, offers a coherent explanation for this pattern. Classes achieving high F1 throughout this study, namely, flood attacks, brute-force password attempts, port and vulnerability scanning, and SQL injection, each succeed through repeated, systematic, or high-volume network action, precisely the kind of behavior flow-level aggregate statistics are well suited to capture. By contrast, MITM, Backdoor, and Ransomware each achieve their objective by minimizing or camouflaging network-visible activity: MITM passively relays already-legitimate traffic; backdoors rely on infrequent, low-profile command-and-control beaconing to evade long-term detection; and ransomware’s primary damage occurs locally, with only sparse, irregular network communication. This distinction between “noisy,” volume-dependent attacks and “low-and-slow,” camouflage-dependent attacks suggests that any detection approach relying on flow-level aggregate statistics, not only the specific 57 features used here, would plausibly face a similar structural limitation against attacks whose entire strategy is to avoid producing such statistics in the first place.

### 5.5. Class-Imbalance Interventions

We evaluated three interventions targeting class imbalance and misclassification among the three most difficult classes: data-level resampling, loss-function design, and classification-architecture restructuring. SMOTE-based oversampling (Section 4.6.1) provided no benefit, modestly reducing F1 for Backdoor, MITM, and Ransomware alike. Focal loss (Section 4.6.2), applied on top of the existing class weights, produced a similar and more severe degradation, with early-training instability; several classes exhibited zero precision and recall in initial checkpoints before recovering over subsequent checkpoints. Restructuring the multiclass head to exclude Normal from its candidate label space (Section 4.6.3) improved Ransomware F1 from 0.379 to 0.435. Backdoor remained largely unresponsive across all three interventions.

Separately, replacing CyberValueCollator’s global-random value substitution with feature-constrained alternatives (zero-replacement or intra-feature swap noise) improved MITM F1 from 0.414 to 0.643 under both variants.

### 5.6. Inference Latency and Deployment Autonomy

The latency comparison in Section 4.5 reveals a genuine tradeoff rather than a straightforward efficiency deficit. CyberRoBERTa’s measured single-flow latency (191 to 240 ms) is substantially slower than both XGBoost’s (1.17 ms) and SEED’s reported end-to-end pipeline (79 ms combined edge-and-device). We do not dispute this gap, but we argue it should be interpreted alongside what CyberRoBERTa’s architecture avoids in exchange: unlike SEED, which achieves its lower latency in part by offloading the computationally intensive embedding-generation step to edge infrastructure, CyberRoBERTa performs the complete detection pipeline, tokenization, transformer encoding, and classification entirely on a single device, with no data transmitted to any external system at any point. This eliminates the network-dependent attack surface and connectivity-reliability concerns that SEED’s authors identify as open limitations of their architecture, and it removes any dependency on edge infrastructure being available, trusted, or uncompromised. We therefore characterize CyberRoBERTa’s higher latency not as an unaddressed weakness but as one side of an explicit architectural tradeoff between inference speed and deployment autonomy: for applications where full on-device operation and minimized network exposure are priorities, for instance, in environments where edge connectivity cannot be guaranteed or where transmitting even derived traffic representations poses an unacceptable privacy or security risk, this tradeoff favors a slower, fully self-contained model over a faster, network-dependent one. We note, however, that at 191 to 240 ms per flow, CyberRoBERTa remains below real-time thresholds appropriate for some but not all IoT monitoring use cases.

### 5.7. Precision-Recall Asymmetry and Operational Considerations

Finally, the binary head’s precision/recall asymmetry, perfect recall and precision on attack and normal traffic, respectively, but attack precision at 94% and normal recall at 92%, is worth interpreting in light of the decision to pretrain exclusively on normal traffic from CICIoT2023 and TON_IoT: although the MLM objective never observes attack traffic or labels during pretraining, having only learned to reconstruct masked values within the statistical distribution of benign behavior leaves the encoder’s internal representation of “expected” traffic constituted entirely by normal patterns, such that, after fine-tuning, flows occupying ambiguous or unfamiliar regions of the feature space are more readily attributed to the attack class than the reverse, since the model’s prior affords a sharper characterization of normal traffic than of any individual attack type. Operationally, this bias toward over-flagging rather than under-flagging is best understood as a genuine deployment tradeoff rather than an unconditional strength. High attack recall is valuable in a setting where missed attacks carry serious consequences, but there is an associated false-positive burden.

## 6. Conclusions

In this study, we present CyberRoBERTa, a cybersecurity-focused small language model pretrained from scratch with a domain-specific tokenizer and data collator, then fine-tuned for intrusion detection in IoT ecosystems. Rather than adapting a general-purpose pretrained model to network traffic after the fact, as is common practice in the literature, we designed a model purpose-built for the constraints of IoT traffic from the outset. We optimize the RoBERTa architecture into a 10.2-million-parameter model, pretrained on normal-traffic partitions of CICIoT2023 and TON_IoT and fine-tuned on a labeled corpus built from Edge-IIoTset. Central contributions include a domain-specific Unigram tokenizer, with 57 locked feature-name tokens, and CyberValueCollator, a masking scheme separating schema tokens from behavioral values; a masking-rate ablation across five configurations showed masking intensity to materially affect downstream performance rather than functioning as an inconsequential hyperparameter. The resulting model achieved a binary classification F1-score of 96% and a multiclass attack classification F1-score of 89.54%.

To further evaluate and test these findings, we conducted five additional experiments. First, we compared CyberRoBERTa against XGBoost, a classical machine learning baseline trained on the same features, and found comparable aggregate accuracy, with CyberRoBERTa’s principal advantage concentrated on MITM, the most data-scarce and behaviorally camouflaged class evaluated. Second, we isolated tokenizer design from truncation and pretraining scale, showing that domain-specific tokenization provides a genuine advantage under matched pretraining conditions; conversely, this advantage is overturned by a fully-scaled, externally-pretrained model, and CyberRoBERTa’s contribution should therefore be understood as achieving competitive performance under the pretraining-scale constraints realistic for a domain-specific model trained from scratch, not as an unconditional advantage over larger models. Third, a model-independent *k*-nearest-neighbor analysis of the retained feature space confirmed that Backdoor, Ransomware, and MITM occupy genuinely overlapping regions independent of any trained classifier, distinguishing attacks that succeed through camouflage from those detectable via aggregate flow statistics. Fourth, we evaluated three interventions targeting class-imbalance and misclassification, namely, SMOTE, focal loss, and a cascaded classification architecture. SMOTE and focal loss did not improve detection, while the cascaded architecture improved Ransomware. Separately, redesigning CyberValueCollator’s value-replacement mechanism, replacing its default global-random substitution with feature-constrained alternatives such as zero-replacement or intra-feature swap noise, significantly improved MITM detection while leaving Backdoor and Ransomware largely unaffected. Fifth, we measured CPU-only latency and memory and conducted a theoretical FLOPs analysis, quantifying CyberRoBERTa’s efficiency without physical edge-device hardware and establishing a projected, though unverified, latency range on Raspberry Pi-class hardware.

Several limitations remain. The first is computational. All model training, pretraining, fine-tuning, the masking-rate ablation, the tokenizer-scale isolation experiments, the classical machine learning baseline’s training, the class-imbalance interventions, and the value-replacement ablation, was conducted on a single NVIDIA Tesla T4 GPU via Google Colaboratory, constraining model scale, batch size, and hyperparameter search alike. Inference latency measurements were separately obtained on a CPU-only runtime specifically to approximate constrained-hardware conditions, this distinction does not remove the underlying computational constraint, since even CPU-only measurement was limited to a single cloud session rather than dedicated, purpose-built edge hardware such as a physical Raspberry Pi. Second, pretraining exclusively on normal traffic leaves the encoder without a prior representation of attack-specific behavior, plausibly contributing the detection ceiling observed for Backdoor and Ransomware, whose difficulty, unlike MITM’s, did not respond to fine-tuning-stage interventions, consistent with a representational gap originating at pretraining rather than one addressable through fine-tuning alone. Moreover this approach leads to alert fatigue, where security personnel begin to discount frequent low-confidence alerts.

The third limitation concerns model scale. This study deliberately evaluates a compact sub-25-million-parameter regime, since IoT deployment was the guiding constraint throughout. Operating at this scale, under the single-GPU computational budget described above, also affects how confidently the reported results can be interpreted: per-class F1 for low-support classes was observed to fluctuate by as much as 0.30 across evaluation checkpoints within a single run, a margin exceeding the differences separating several configurations compared in this study. Separately, the differences we report between domain-specific and generic tokenization, and between CyberValueCollator variants, are consequently observed at small scale, where such differences tend to be more pronounced; whether they persist, strengthen, or diminish at the scale of larger language models remains untested here and, given the deployment goals of this work, was considered out of scope. We view this as a reasonable scope restriction rather than an oversight, though a natural direction for future work is to test whether the design principles introduced here, namely, domain-specific tokenization and schema-aware masking, transfer to larger models when constrained deployment is not the primary objective, for instance, in cloud-based or server-side threat-detection settings where greater computational resources are available.

The fourth limitation follows directly from CyberRoBERTa’s central design choice: performing the complete detection pipeline on a single, self-contained device, with no computation offloaded elsewhere. This autonomy comes at a real cost. Because the encoder must carry the full computational burden itself, both CyberRoBERTa’s model size and its measured inference latency are larger than what is typical for the most aggressively lightweight IoT solutions, for instance, embedding-offloading architectures or classical models such as XGBoost, both of which achieve smaller on-device footprints and faster inference precisely because they delegate part of the workload elsewhere or avoid dense neural computation altogether. We treat this as an explicit tradeoff rather than an unaddressed weakness, but it remains a genuine limitation for deployment contexts where even CyberRoBERTa’s compact footprint or measured latency exceed what available hardware or application timing requirements can accommodate.

Future work will pursue several directions. First, finer-grained masking-rate and corruption-strategy sweeps. Second, evaluation on additional IoT datasets to assess generalization beyond Edge-IIoTset. Third, a systematic hyperparameter search. Fourth, evaluating whether CyberRoBERTa’s pretraining-derived prior offers a genuine advantage under label-scarce conditions. The comparison against XGBoost in this study (Section 4.5) was conducted using the full labeled fine-tuning corpus, under which XGBoost performed competitively with, and in several respects exceeded, CyberRoBERTa. XGBoost, however, learns exclusively from labeled data, with no mechanism for leveraging unlabeled traffic, whereas CyberRoBERTa’s pretraining stage builds a general representation of normal IoT behavior from an unlabeled corpus before any labeled supervision is introduced. Evaluating both models as labeled examples per attack type are progressively reduced would test whether this pretraining-derived prior provides a measurable advantage under label scarcity, a realistic scenario for new deployment environments or newly emerging attack types where large labeled datasets are not yet available. Fifth, a class-adaptive corruption strategy for CyberValueCollator, applying feature-constrained substitution such as zero-replacement or intra-feature swap noise specifically to severely underrepresented classes while retaining global-random substitution elsewhere, motivated directly by the class-specific pattern observed in this study’s value-replacement ablation. Sixth, model quantization and an optimized inference runtime, to narrow the gap between CyberRoBERTa’s measured and projected latency without reintroducing a network dependency. Seventh, testing whether the domain-specific tokenization and schema-aware masking principles introduced here transfer to larger-scale models, a question this study deliberately leaves untested given its IoT deployment focus, Eighth, testing different masking strategies, such as pure masking or dynamic masking.

## Figures and Tables

**Table 1:** Selected IoT datasets used for pretraining and fine-tuning.

Dataset	Role	Size (MB)	Year
CICIoT2023	Pretraining	197 ^1^	2023
TON_IoT	Pretraining	197 ^1^	2020
Edge-IIoTset	Fine-tuning	66.8	2022

^1^ 197 MB is the combined size of the pretraining corpus drawn from the normal-traffic partitions of both CICIoT2023 and TON_IoT (i.e., not two independent 197 MB corpora). All datasets are publicly available.

**Table 2:** Dropped features and leakage justification.

Feature(s)	Leakage Group	Why Dropped
id, {expiration,node}_id	Pure identifiers	Encodes dataset structure, not attack behavior.
{src,dst}_{ip,mac,oui}	Machine identity	Identifies the source, not the attack; fixed attacker addresses would be memorized rather than generalized.
{bidirectional,src2dst,dst2src}_{first,last}_seen_ms	Timestamps	Encodes when attacks were injected, not what the attack looks like.
requested_server_name, client_fingerprint, user_agent, content_type	Tool-specific signals	Identifies the attack tool, not the attack class.
application_{name,category_name,is_guessed,confidence}	DPI/label circularity	May indirectly encode the ground-truth label.

**Table 3:** Retained features by group and dimension.

Group	Dimension	Features
Bidirectional	Packet count	bidirectional_packets
	Byte count	bidirectional_bytes
	Duration	bidirectional_duration_ms
	TCP flags	bidirectional_{syn,fin,rst,psh,ack,urg,cwr,ece}_packets
	Inter-arrival time	bidirectional_{min,max,mean,stddev}_piat_ms
	Packet size	bidirectional_{min,max,mean,stddev}_ps
Src→Dst	Packet count	src2dst_packets
	Byte count	src2dst_bytes
	Duration	src2dst_duration_ms
	TCP flags	src2dst_{syn,fin,rst,psh,ack,urg,cwr,ece}_packets
	Inter-arrival time	src2dst_{min,max,mean,stddev}_piat_ms
	Packet size	src2dst_{min,max,mean,stddev}_ps
Dst→Src	Packet count	dst2src_packets
	Byte count	dst2src_bytes
	Duration	dst2src_duration_ms
	TCP flags	dst2src_{syn,fin,rst,psh,ack,urg,cwr,ece}_packets
	Inter-arrival time	dst2src_{min,max,mean,stddev}_piat_ms
	Packet size	dst2src_{min,max,mean,stddev}_ps

**Table 4:** Per-class F1-scores across masking configurations.

Class	15% Std	15% CVC	20% CVC	25% CVC	30% CVC	Best Config
Backdoor	0.6331	**0.6481**	0.6478	0.6350	0.6324	15% CVC
HTTP_Flood	0.9739	0.9735	**0.9933**	0.9868	0.9735	20% CVC
MITM	0.4444	**0.4571**	0.4138	0.4286	0.3636	15% CVC
Normal	**0.9598**	0.9586	0.9581	0.9586	0.9577	15% Std
Password	**0.9892**	0.9781	0.9855	**0.9892**	0.9819	15% Std/25% CVC
Port_Scanning	**0.9742**	**0.9742**	0.9670	**0.9742**	**0.9742**	all except 20% CVC
Ransomware	0.3212	0.3168	**0.3956**	0.3301	0.3278	20% CVC
SQL_Injection	**1.0000**	**1.0000**	**1.0000**	**1.0000**	**1.0000**	all
Scanning_Vuln	0.9597	0.9231	**0.9635**	**0.9635**	0.9562	20% CVC/25% CVC
TCP_SYN_Flood	**1.0000**	**1.0000**	**1.0000**	**1.0000**	**1.0000**	all
UDP_Flood	**1.0000**	**1.0000**	**1.0000**	**1.0000**	**1.0000**	all
XSS	0.7782	0.7368	**0.7812**	0.7687	0.7638	20% CVC
Macro Avg	0.8361	0.8305	**0.8422**	0.8362	0.8276	20% CVC

CVC: CyberValueCollator with a 10-10-80 corruption scheme, numerical-only masking. Std: standard HuggingFace data collator. Bold indicates the best F1 per class.

**Table 5:** Per-class F1 across CyberValueCollator value-replacement strategies.

Class	Global-Random	Swap (Intra-Feature)	Zero-Replacement
Backdoor	**0.6478**	0.5360	0.5882
HTTP_Flood	**0.9933**	0.9799	0.9799
MITM	0.4138	**0.6429**	**0.6429**
Normal	0.9581	**0.9641**	**0.9641**
Password	0.9855	0.9730	**0.9865**
Port_Scanning	**0.9670**	0.9404	0.9437
Ransomware	**0.3956**	0.3803	0.3442
SQL_Injection	**1.0000**	**1.0000**	**1.0000**
Scanning_vulnerability	**0.9635**	0.9306	0.9306
TCP_SYN_Flood	**1.0000**	**1.0000**	0.9967
UDP_Flood	**1.0000**	**1.0000**	**1.0000**
XSS	0.7812	0.7752	**0.7905**
Macro F1	0.8422	0.8435	**0.8473**

Bold indicates the best F1 per class (ties are all shown in bold).

**Table 6:** Tokenizer comparison: domain-specific (Unigram) vs. generic (roberta-base).

Metric	Domain-Specific	Generic (Roberta-Base)
Feature names preserved intact	4/4	0/4
Mean sequence length (tokens)	253.58	928.87
95th percentile length	301	958
Vocabulary utilization	95.60%	2.87%
Unknown-token rate	0.00%	0.00%
Roundtrip fidelity	100%	100%

**Table 7:** Macro F1 across tokenizer, truncation, and pretraining-scale conditions.

Model	Params	Pretraining	Macro F1
CyberRoBERTa (domain tokenizer)	10.2 M	From scratch, IoT corpus (∼197 MB)	0.8422
Generic tokenizer, from scratch	24.0 M	From scratch, same IoT corpus	0.8366
roberta-base, truncated (512)	125 M	Large-scale external text pretraining	0.8487
roberta-base, extended (∼1024)	125 M	Large-scale external text pretraining	0.8468

**Table 8:** Binary classification results (2639 test flows).

Class	Precision	Recall	F1	Support
Normal (0)	1.00	0.92	0.96	1178
Attack (1)	0.94	1.00	0.97	1461
Macro average	0.97	0.96	0.96	2639
Weighted average	0.97	0.96	0.96	2639
Overall F1-score			0.96	2639

**Table 9:** Multiclass classification results (2639 test flows).

Attack Class	Precision	Recall	F1	Support
Backdoor	0.7407	0.5755	0.6478	139
HTTP Flood	1.0000	0.9868	0.9933	151
MITM	0.8571	0.2727	0.4138	22
Normal	0.9963	0.9228	0.9581	1178
Password	1.0000	0.9714	0.9855	140
Port scanning	0.9851	0.9496	0.9670	139
Ransomware	0.2697	0.7417	0.3956	120
SQL injection	1.0000	1.0000	1.0000	138
Scanning vulnerability	1.0000	0.9296	0.9635	142
TCP SYN flood	1.0000	1.0000	1.0000	160
UDP flood	1.0000	1.0000	1.0000	154
XSS	1.0000	0.6410	0.7812	156
Macro average	0.9041	0.8326	0.8422	2639
Weighted average	0.9495	0.8954	0.9126	2639
Overall F1-score			0.8954	2639

**Table 10:** XGBoost binary classification results (2639 test flows).

Class	Precision	Recall	F1	Support
Normal (0)	1.00	0.94	0.97	1154
Attack (1)	0.95	1.00	0.98	1485
Macro average	0.98	0.97	0.97	2639
Weighted average	0.97	0.97	0.97	2639
Overall F1-score			0.97	2639
ROC AUC			0.9956	

**Table 11:** XGBoost multiclass classification results (2639 test flows).

Attack Class	Precision	Recall	F1	Support
Backdoor	0.65	0.53	0.59	135
HTTP Flood	0.99	0.99	0.99	150
MITM	0.11	0.53	0.19	19
Normal	1.00	0.94	0.97	1154
Password	1.00	0.98	0.99	150
Port Scanning	1.00	0.95	0.97	150
Ransomware	0.37	0.60	0.46	131
SQL Injection	1.00	1.00	1.00	150
Scanning Vulnerability	0.97	0.91	0.94	150
TCP SYN Flood	1.00	1.00	1.00	150
UDP Flood	1.00	1.00	1.00	150
XSS	0.94	0.72	0.82	150
Macro average	0.84	0.85	0.83	2639
Weighted average	0.94	0.90	0.91	2639
Overall F1-score			0.90	2639

**Table 12:** Effect of SMOTE oversampling on per-class F1.

Class	Baseline F1	SMOTE F1	Δ
Backdoor	0.6478	0.5878	−0.0099
MITM	0.4138	0.4118	−0.0327
Ransomware	0.3956	0.3284	−0.0507

**Table 13:** Effect of focal loss on per-class F1.

Class	Baseline F1	Focal Loss F1	Δ
Backdoor	0.6478	0.6426	+0.0449
MITM	0.4138	0.2667	−0.1777
Ransomware	0.3956	0.3478	−0.0313

**Table 14:** Effect of cascaded (11-way, Normal-excluded) architecture on per-class F1.

Class	Baseline F1 (12-Way)	Cascaded F1 (11-Way)	Δ
Backdoor	0.6478	0.5869	−0.0108
MITM	0.4138	0.5714	+0.1270
Ransomware	0.3956	0.4348	+0.0557

**Table 15:** *k*-nearest-neighbor (k=10) label purity by class.

Class	Purity
Backdoor	0.370
HTTP_Flood	0.989
MITM	0.224
Normal	0.921
Password	0.981
Port_Scanning	0.929
Ransomware	0.438
SQL_Injection	1.000
Scanning_vulnerability	0.912
TCP_SYN_Flood	1.000
UDP_Flood	1.000
XSS	0.656

## Data Availability

The processed datasets presented in this study are available on Hugging Face at https://huggingface.co/datasets/dhai1999/small-model-roberta (accessed on 2 October 2026), and the pretrained model weights are available at https://huggingface.co/dhai1999/small-model-roberta-models (accessed on 2 October 2026). These data were derived from the following resources available in the public domain: CICIoT2023 (https://www.unb.ca/cic/datasets/iotdataset-2023.html (accessed on 2 October 2026), DOI: 10.3390/s23135941), TON_IoT (https://research.unsw.edu.au/projects/toniot-datasets (accessed on 2 October 2026), DOI: 10.1109/ACCESS.2020.3022862), and Edge-IIoTset (https://www.kaggle.com/datasets/mohamedamineferrag/edgeiiotset-cyber-security-dataset-of-iot-iiot (accessed on 2 October 2026), DOI: 10.1109/ACCESS.2022.3165809).

## Abbreviations

The following abbreviations are used in this manuscript:

AE	Autoencoder
APT	Advanced Persistent Threat
AUC	Area Under the Curve
BERT	Bidirectional Encoder Representations from Transformers
CIC	Canadian Institute for Cybersecurity
CNN	Convolutional Neural Network
CVC	CyberValueCollator
DDoS	Distributed Denial-of-Service
DL	Deep Learning
DoS	Denial-of-Service
DPI	Deep Packet Inspection
DT	Decision Tree
FP16	16-bit Floating-Point (Mixed-Precision Training)
GA	Genetic Algorithm
GPT	Generative Pretrained Transformer
GPU	Graphics Processing Unit
HTTP	HyperText Transfer Protocol
IDS	Intrusion Detection System
IG	Information Gain
IIoT	Industrial Internet of Things
IoT	Internet of Things
IoV	Internet of Vehicles
KNN	K-Nearest Neighbors
LLM	Large Language Model
LR	Logistic Regression
LSTM	Long Short-Term Memory
ML	Machine Learning
MITM	Man-in-the-Middle
MLM	Masked Language Modeling
NFKC	Unicode Normalization Form Compatibility Composition
NL	Natural Language
PCA	Principal Component Analysis
PPFLE	Privacy-Preserving Fixed-Length Encoding
RF	Random Forest
ROC	Receiver Operating Characteristic
SEIT	School of Engineering and Information Technology
SNI	Server Name Indication
SQL	Structured Query Language
Std	Standard (HuggingFace Data Collator Baseline)
T5	Text-to-Text Transfer Transformer
TCP	Transmission Control Protocol
TLS	Transport Layer Security
UDP	User Datagram Protocol
VRAM	Video Random Access Memory
XGB	Extreme Gradient Boosting
XSS	Cross-Site Scripting

## References

[1] Jameel M., Chowdhary Y. (2026). Internet of Things for Smart Cities: Technologies, Applications and Challenges. Int. J. Multidiscip. Res. (IJFMR).

[2] Li C., Wang J., Wang S., Zhang Y. (2024). A review of IoT applications in healthcare. Neurocomputing.

[3] Qiu F., Kumar A., Hu J., Sharma P., Tang Y.B., Xiang Y.X., Hong J. (2025). A Review on Integrating IoT, IIoT, and Industry 4.0: A Pathway to Smart Manufacturing and Digital Transformation. IET Inf. Secur..

[4] Vardakis G., Hatzivasilis G., Koutsaki E., Papadakis N. (2024). Review of Smart-Home Security Using the Internet of Things. Electronics.

[5] IoT Analytics (2025). State of IoT 2025: Number of Connected IoT Devices Growing 14% to 21.1 Billion Globally. https://iot-analytics.com/number-connected-iot-devices/.

[6] Kabir M.A.A., Elmedany W., Sharif M.S. (2023). Securing IoT Devices Against Emerging Security Threats: Challenges and Mitigation Techniques. J. Cyber Secur. Technol..

[7] Kelechukwu Madu V. (2025). Security Challenges in IoT: A Systematic Literature Review. Eng. Technol. J..

[8] Feng Y., Sakurai K. (2025). Network Intrusion Detection: Evolution from Conventional Approaches to LLM Collaboration and Emerging Risks. arXiv.

[9] Musthafa D. (2025). Real-Time Intrusion Detection Leveraging Deep Learning: A Comparative Analysis of CNN, RNN, and Transformer Architectures. Int. J. Adv. Eng. Manag. Sci..

[10] Chkirbene Z., Hamila R., Gouissem A., Devrim U. (2024). Large Language Models (LLM) in Industry: A Survey of Applications, Challenges, and Trends. Proceedings of the 2024 IEEE 21st International Conference on Smart Communities: Improving Quality of Life using AI, Robotics and IoT (HONET).

[11] Jaffal N.O., Alkhanafseh M., Mohaisen D. (2025). Large Language Models in Cybersecurity: A Survey of Applications, Vulnerabilities, and Defense Techniques. AI.

[12] Diaf A., Korba A.A., Elislem Karabadji N., Ghamri-Doudane Y. (2024). Beyond Detection: Leveraging Large Language Models for Cyber Attack Prediction in IoT Networks. Proceedings of the 2024 20th International Conference on Distributed Computing in Smart Systems and the Internet of Things (DCOSS-IoT), Los Alamitos, CA, USA, 2024.

[13] Fu M., Wang P., Liu M., Zhang Z., Zhou X. (2025). IoV-BERT-IDS: Hybrid Network Intrusion Detection System in IoV Using Large Language Models. IEEE Trans. Veh. Technol..

[14] Ferrag M.A., Ndhlovu M., Tihanyi N., Cordeiro L.C., Debbah M., Lestable T., Thandi N.S. (2024). Revolutionizing Cyber Threat Detection With Large Language Models: A Privacy-Preserving BERT-Based Lightweight Model for IoT/IIoT Devices. IEEE Access.

[15] Chowdhury S., Purushotham E., Srinivasan A., Sreeraman Y. (2024). Predicting Cyber-Attacks Through the Use of Deep Learning Algorithms. Proceedings of the 2024 5th International Conference on Innovative Trends in Information Technology (ICITIIT).

[16] Sattarpour S., Barati A., Barati H. (2024). EBIDS: Efficient BERT-based intrusion detection system in the network and application layers of IoT. Clust. Comput..

[17] Bommarito M.J., Katz D.M., Bommarito J. (2025). KL3M Tokenizers: A Family of Domain-Specific and Character-Level Tokenizers for Legal, Financial, and Preprocessing Applications. arXiv.

[18] Li Q., Tong J., Liu S., Li C., Tang J., Zhou Q. (2026). Medical knowledge representation enhancement in large language models through clinical tokens optimization. Sci. Rep..

[19] Afifi F., Zaki F., Hanif H., Aqil N., Anuar N. (2025). Transformer-based tokenization for IoT traffic classification across diverse network environments. PeerJ Comput. Sci..

[20] Wettig A., Gao T., Zhong Z., Chen D., Vlachos A., Augenstein I. (2023). Should You Mask 15% in Masked Language Modeling?. Proceedings of the 17th Conference of the European Chapter of the Association for Computational Linguistics.

[21] Warner B., Chaffin A., Clavié B., Weller O., Hallström O., Taghadouini S., Gallagher A., Biswas R., Ladhak F., Aarsen T., Che W., Nabende J., Shutova E., Pilehvar M.T. (2025). Smarter, Better, Faster, Longer: A Modern Bidirectional Encoder for Fast, Memory Efficient, and Long Context Finetuning and Inference. Proceedings of the 63rd Annual Meeting of the Association for Computational Linguistics (Volume 1: Long Papers).

[22] Alrefaei A., Ilyas M. (2024). Using Machine Learning Multiclass Classification Technique to Detect IoT Attacks in Real Time. Sensors.

[23] Craciun R., Caramihai S.I., Mocanu S.A., Pietraru R.N., Moisescu M.A. (2025). Hybrid Machine Learning for IoT-Enabled Smart Buildings. Informatics.

[24] Chen J., Xiao J., Xu J. (2024). VGGIncepNet: Enhancing Network Intrusion Detection and Network Security through Non-Image-to-Image Conversion and Deep Learning. Electronics.

[25] Hazman C., Guezzaz A., Benkirane S., Azrour M. (2024). Enhanced IDS with Deep Learning for IoT-Based Smart Cities Security. Tsinghua Sci. Technol..

[26] Adjewa F., Esseghir M., Merghem-Boulahia L. (2026). From Edge Transformer to IoT Decisions: Offloaded Embeddings for Lightweight Intrusion Detection. Sensors.

[27] Guo R., Li A., Liu H. (2025). An Adversarial Attack Detection Method Based on Bidirectional Consistency Discrimination for Deep Learning-Based Soft Sensors. Proceedings of the 2025 CAA Symposium on Fault Detection, Supervision, and Safety for Technical Processes (SAFEPROCESS), Urumqi, China, 22–24 August 2025.

[28] Akuthota U.C., Bhargava L. (2025). Transformer-Based Intrusion Detection for IoT Networks. IEEE Internet Things J..

[29] Iida H., Thai D., Manjunatha V., Iyyer M., Toutanova K., Rumshisky A., Zettlemoyer L., Hakkani-Tur D., Beltagy I., Bethard S., Cotterell R., Chakraborty T., Zhou Y. (2021). TABBIE: Pretrained Representations of Tabular Data. Proceedings of the 2021 Conference of the North American Chapter of the Association for Computational Linguistics: Human Language Technologies.

[30] Neto E.C.P., Dadkhah S., Ferreira R., Zohourian A., Lu R., Ghorbani A.A. (2023). CICIoT2023: A Real-Time Dataset and Benchmark for Large-Scale Attacks in IoT Environment. Sensors.

[31] Alsaedi A., Moustafa N., Tari Z., Mahmood A.N., Anwar A. (2020). TON_IoT Telemetry Dataset: A New Generation Dataset of IoT and IIoT for Data-Driven Intrusion Detection Systems. IEEE Access.

[32] Ferrag M.A., Friha O., Hamouda D., Maglaras L., Janicke H. (2022). Edge-IIoTset: A New Comprehensive Realistic Cyber Security Dataset of IoT and IIoT Applications: Centralized and Federated Learning. IEEE Access.

[33] Aouini Z., Pekar A. (2022). NFStream: A flexible network data analysis framework. Comput. Netw..

[34] Lin T., Goyal P., Girshick R.B., He K., Dollár P. (2017). Focal Loss for Dense Object Detection. arXiv.

[35] Grinsztajn L., Oyallon E., Varoquaux G. (2022). Why do tree-based models still outperform deep learning on tabular data?. arXiv.

